# *CircUBAP2(9,12)* Inhibits Nasopharyngeal Carcinoma Invasion and Metastasis by Down-regulating *ZEB2* through Competitive Binding to AUF1

**DOI:** 10.34133/research.0936

**Published:** 2025-11-04

**Authors:** Chunmei Fan, Zhiqi Guo, Hongke Qu, Qijia Yan, Dan Wang, Yumin Wang, Shanshan Zhang, Can Guo, Zhaoyang Zeng, Wei Xiong, Lei Shi

**Affiliations:** ^1^ NHC Key Laboratory of Carcinogenesis and Hunan Key Laboratory of Cancer Metabolism, Hunan Cancer Hospital and the Affiliated Cancer Hospital of Xiangya School of Medicine, Central South University, Changsha 410013, China.; ^2^ Department of Histology and Embryology, Xiangya School of Medicine, Central South University, Changsha 410013, China.; ^3^ Key Laboratory of Carcinogenesis and Cancer Invasion of the Chinese Ministry of Education, Cancer Research Institute and Xiangya School of Basic Medical Sciences, Central South University, Changsha 410078, China.; ^4^ Department of Otolaryngology Head and Neck Surgery, Xiangya Hospital, Central South University, Changsha 410078, China.; ^5^ Department of Stomatology, Xiangya Hospital, Central South University, Changsha 410078, China.; ^6^ FuRong Laboratory, Changsha 410078, China.; ^7^ National Engineering Research Center of Personalized Diagnostic and Therapeutic Technology, Central South University, Changsha 410008, China.; ^8^ Department of Pathology, The Second Xiangya Hospital, Central South University, Changsha 410011, China.

## Abstract

Nasopharyngeal carcinoma (NPC) is identified as a highly aggressive epithelial malignancy. Despite advances in therapy, metastatic relapse remains the leading cause of mortality, underscoring the urgent need for novel molecular targets. Although circular RNAs participate in tumor initiation and progression, their biological functions and internal mechanisms in NPC metastasis remain largely undefined. In this study, we identified a novel circular RNA, *circUBAP2(9,12)*, generated by back-splicing of exons 9 to 12 of the ubiquitin-associated protein 2 (*UBAP2*) pre-mRNA. *CircUBAP2(9,12)* was found to be notably down-regulated in NPC tissues, and its low expression was associated with unfavorable prognosis. Functional assays demonstrated that *circUBAP2(9,12)* suppresses NPC cell migration and metastasis both in vitro and in vivo. Mechanistically, *circUBAP2(9,12)* competitively binds to the RNA-binding protein adenylate-uridylate-rich binding factor 1 (AUF1), thereby reducing AUF1’s interaction with zinc finger E-box binding homeobox 2 (*ZEB2*) mRNA. This leads to decreased stability of *ZEB2* mRNA, inhibition of the epithelial–mesenchymal transition, and consequent suppression of NPC metastasis. Overall, this study reveals a critical role of *circUBAP2(9,12)* in NPC progression and highlights its potential as a therapeutic target for metastatic NPC.

## Introduction

Nasopharyngeal carcinoma (NPC) displays a pronounced geographical distribution, with notably increased prevalence in Southern China, Southeast Asia, and Northern Africa [1]. Epidemiological data indicate that the incidence in these regions ranges from 4 to 25 cases per 100,000 individuals—approximately 50- to 100-fold higher than in other parts of the world [2]. This distinct regional clustering suggests a multifactorial etiology including genetic susceptibility background, persistent Epstein–Barr virus involvement, and region-specific environmental exposures [3]. The insidious onset of NPC contributes to substantial diagnostic delays, as most people are diagnosed at advanced stages. This is largely attributed to the concealed location of the nasopharynx and the nonspecific nature of early symptoms [4]. Although advances in intensity-modulated radiotherapy have improved outcomes, the survival percentage over 5 years for metastatic NPC remains below 50%, with treatment failure mainly due to distant metastasis and locoregional recurrence [5,6]. In this sense, delineating the mechanistic underpinnings of NPC progression and discovering druggable molecules are critical for improving diagnosis and treatment outcomes.

Circular RNAs (circRNAs) are noncoding RNAs formed through back-splicing of precursor messenger RNAs (pre-mRNAs) [7]. CircRNAs were identified as important epigenetic regulators, with their dysregulation implicated in a variety of diseases [8], including cancer, highlighting their potential as druggable targets [9–12]. While circRNAs are primarily generated via back-splicing [13], some also influence the splicing of their linear precursors [14,15]. CircRNAs have been shown to contribute to tumor progression through multiple mechanisms, including acting as microRNA (miRNA) sponges [16–18], participating in epigenetic regulation [19], and modulating protein posttranslational modifications [20]. Notably, accumulating evidence indicates that certain circRNAs can serve as protein decoys or scaffolds, directly regulating the activity of RNA-binding proteins (RBPs)—a paradigm-shifting mechanism that remains largely unexplored in NPC. Specific circRNAs harbor RBP-binding sites [21], or open reading frames, enabling them to interact with RBPs or even encode functional peptides [22]. Despite their well-established roles in other malignancies, the biological significance of circRNA–RBP interactions in NPC metastasis remains an important and underexplored area of research.

To date, more than 1,500 RBPs have been identified in humans, and dysregulation of their expression is involved in cancer progression [23]. Among them, adenylate-uridylate-rich binding factor 1 (AUF1) is a key posttranscriptional regulator that modulates gene expression by binding to adenylate-uridylate-rich elements (AREs) in the 3′ untranslated regions (3′UTRs) [24], thereby influencing their stability and degradation rates [25]. Despite its well-established role in mRNA decay, the regulatory mechanisms governing AUF1 activity in NPC, particularly those involving circRNA-mediated interactions, remain poorly understood. This gap in knowledge presents a compelling opportunity for uncovering novel molecular pathways underlying NPC progression.

In this study, we identified *circUBAP2(9,12)* as a previously uncharacterized circRNA derived from exons 9 to 12 of the ubiquitin-associated protein 2 (*UBAP2*) pre-mRNA, distinct from other reported *UBAP2*-derived circRNAs. *CircUBAP2(9,12)* is markedly lowly expressed in NPC tissues and functions as a suppressor of NPC cell migration and metastasis. Mechanistically, *circUBAP2(9,12)* competitively binds to the RBP protein AUF1, thereby reducing AUF1’s association with zinc finger E-box binding homeobox 2 (*ZEB2*) mRNA. This interaction leads to decreased stability of *ZEB2* mRNA, resulting in inhibition of the epithelial–mesenchymal transition (EMT) in NPC cells. Collectively, our findings expand the functional repertoire of *circUBAP2(9,12)* in cancer and identify AUF1 as a previously unrecognized regulator of NPC progression. This study provides compelling evidence that circRNAs can modulate mRNA stability by directly competing with RBPs and establishes the *circUBAP2(9,12)*–AUF1–ZEB2 axis as a clinically relevant regulatory pathway. These insights decidedly advance our understanding of circRNA-mediated posttranscriptional regulation in cancer metastasis and establishing mechanistic insights for NPC therapeutics.

## Results

### *CircUBAP2(9,12)* is down-regulated in NPC and its reduced expression is associated with unfavorable prognosis

**Fig. 1.**
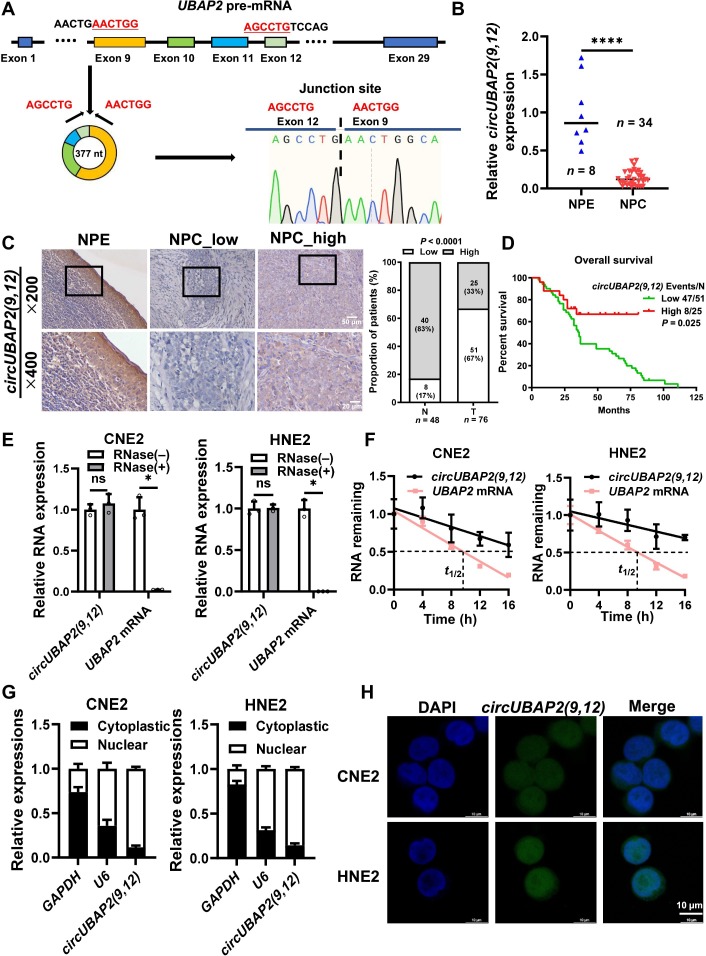
*circUBAP2(9,12)* is down-regulated in nasopharyngeal carcinoma (NPC), and its reduced expression is associated with unfavorable prognosis. (A) Schematic illustration of *circUBAP2(9,12)* loop formation. Sanger sequencing confirmed that *circUBAP2(9,12)* is generated by back-splicing of exons 9 to 12 of *UBAP2* pre-messenger RNA (pre-mRNA). (B) Quantitative real-time polymerase chain reaction (qRT-PCR) analysis showing reduced *circUBAP2(9,12)* expression in NPC tissues (*n* = 34) compared to that in normal nasopharyngeal epithelium (NPE) controls (*n* = 8). (C) In situ hybridization assays of *circUBAP2(9,12)* in 76 NPC paraffin-embedded tissue sections and 48 adjacent normal epithelial tissues. Representative images (left) show spatial distribution at ×200 (scale bar = 50 μm) and ×400 magnification (scale bar = 20 μm). Quantification (right) demonstrates lower *circUBAP2(9,12)* expression in NPC specimens compared to that in controls. (D) Kaplan–Meier survival analysis was performed for *circUBAP2(9,12)* expression in 76 patients with NPC. (E) Stability of *circUBAP2(9,12)* and *UBAP2* mRNA was detected by RNase R treatment followed by qRT-PCR in CNE2 and HNE2 cells. (F) Stability of *circUBAP2(9,12)* and *UBAP2* mRNA was assessed by actinomycin D treatment followed by qRT-PCR in CNE2 and HNE2 cells. (G) Nuclear–cytoplasmic fractionation was performed to assess the localization of *circUBAP2(9,12)*, contrasting with nuclear U6 and cytoplasmic glyceraldehyde-3-phosphate dehydrogenase (GAPDH) controls [42]. (H) Fluorescence in situ hybridization confirmed nuclear enrichment of *circUBAP2(9,12)* (green) in NPC cells, with nuclei counterstained by 4′,6-diamidino-2-phenylindole (DAPI; blue). Scale bar = 10 μm. Results are shown as mean ± standard deviation from a minimum of 3 separate experimental repetitions. ns, not significant; *P < 0.05; ****P < 0.0001.

To identify dysregulated circRNAs involved in NPC, we analyzed circRNA RNA sequencing data from NPC cell lines CNE2 and SUNE1, as well as the immortalized nasopharyngeal cell line NP69 (normal control), downloaded from GSE181906. Among the top 10 significantly down-regulated circRNAs, *has_circ_0001847*—derived from the *UBAP2* gene—stood out (Fig. S1A). While several circRNA isoforms from *UBAP2* have been reported, this particular circular form, which we named *circUBAP2(9,12)*, has not been described previously and showed the highest expression abundance among *UBAP2*-derived variants (Table S7). We selected *has_circ_0003496* (formed by the circularization of exons 7 and 8 of *UBAP2*) and *has_circ_0003141* (formed by the circularization of exons 7 to 12 of *UBAP2*) and compared their expression with that of *circUBAP2 (9,12)* in the normal nasopharyngeal epithelial cell (NP69) and NPC cells (CNE2 and HNE2). The results showed that the expression of *circUBAP2 (9,12)* was lower in NPC cells than in normal cells, and the expression of *has_circ_0003496* and *has_circ_0003141* was even lower, with no significant difference between NPC cells and normal cells (Fig. S1B). According to circBase, *circUBAP2(9,12)* arises from canonical back-splicing of exons 9 to 12 of the *UBAP2* pre-mRNA. We validated its specific circular form and back-splicing junction by quantitative real-time polymerase chain reaction (qRT-PCR) combined with Sanger sequencing, confirming a 377-nt transcript formed by precise joining of exons 9 to 12 (Fig. 1A). We measured *circUBAP2(9,12)* expression in 34 NPC tissues and 8 chronic inflammatory controls, revealing notable down-regulation in NPC compared to noncancerous tissues (Fig. 1B).

To further verify its expression and prognostic relevance, in situ hybridization (ISH) was performed on an independent cohort of 76 NPC samples and 48 adjacent normal epithelial tissues, confirming low *circUBAP2(9,12)* expression in tumors (Fig. 1C). Importantly, patients with low *circUBAP2(9,12)* expression demonstrated obviously shorter overall survival than high-expression counterparts, as determined by Kaplan–Meier analysis (Fig. 1D), indicating that reduced *circUBAP2(9,12)* correlates with poor prognosis. Functionally, *circUBAP2(9,12)* exhibited stability against RNase R degradation, whereas linear *UBAP2* mRNA was nearly completely degraded, confirming its covalently closed circular structure (Fig. 1E). Moreover, after treatment of NPC cells with actinomycin D, *circUBAP2(9,12)* exhibited a substantially longer half-life than linear *UBAP2* mRNA (Fig. 1F). Subcellular fractionation and fluorescence ISH assays showed that *circUBAP2(9,12)* is localized to both the nucleus and cytoplasm, with predominant nuclear distribution (Fig. 1G and H). Collectively, these findings support a tumor-suppressive role for *circUBAP2(9,12)* in NPC pathogenesis.

### *CircUBAP2(9,12)* suppresses the migration and invasion of NPC cells in vitro

**Fig. 2.**
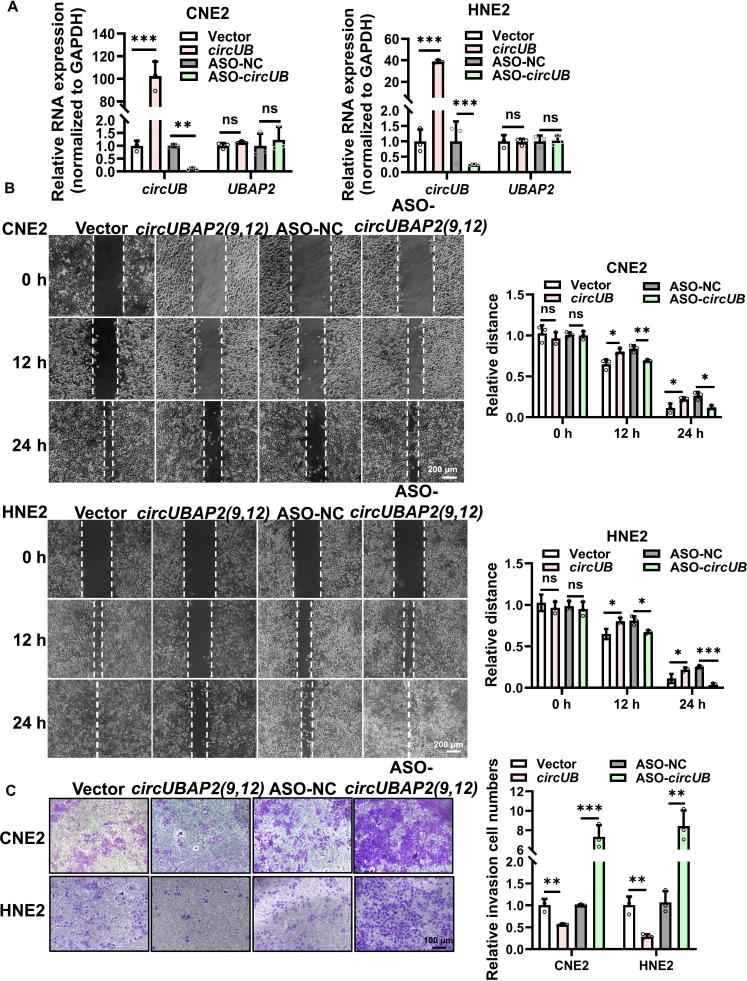
*circUBAP2(9,12)* suppresses the migration and invasion of NPC cells in vitro. (A) *CircUBAP2(9,12)* and its linear counterpart *UBAP2* expression levels were quantified by qRT-PCR following overexpression or knockdown of *circUBAP2(9,12)*. (B) Wound healing assays were conducted to evaluate the migratory ability of CNE2 and HNE2 cells after *circUBAP2(9,12)* overexpression or knockdown. Scale bar = 200 μm. (C) Transwell assays were employed to measure the invasive capacities of NPC cells following overexpression or knockdown of *circUBAP2(9,12)*. Scale bar = 100 μm. Results are shown as mean ± standard deviation from a minimum of 3 separate biological repetitions. ns, not significant; *P < 0.05; **P < 0.01; ***P < 0.001. *circUB*, *circUBAP2(9,12)*; ASO-NC, negative control antisense oligonucleotide (ASO); ASO-*circUB*, ASO-*circUBAP2(9,12)*.

To further investigate the biological function of *circUBAP2(9,12)* in NPC, we designed antisense oligonucleotide (ASO)-*circUBAP2(9,12)* targeting its back-splice junction to achieve specific silencing. Concurrently, an overexpression vector harboring *circUBAP2(9,12)* was successfully constructed. Post-transfection, qRT-PCR confirmed efficient overexpression or knockdown of *circUBAP2(9,12)* (Fig. 2A), without altering the expression of the parent *UBAP2* mRNA. Given that metastasis and recurrence are the primary causes [26] of treatment failure in NPC, we examined whether *circUBAP2(9,12)* affects the migratory and invasive capacities of NPC cells. Wound healing and transwell assays demonstrated that *circUBAP2(9,12)* overexpression remarkably inhibited migration and invasion, whereas its silencing yielded reciprocal effect (Fig. 2B and C and Fig. S1C and D). These results position that *circUBAP2(9,12)* suppresses malignant progression in NPC by impairing cell migration and invasion. Additionally, the potential coding capacity of *circUBAP2(9,12)* was assessed using circRNADb [27], which revealed no open reading frame (Fig. S1E), suggesting that *circUBAP2(9,12)* exerts its function through noncoding mechanisms rather than peptide translation.

### *CircUBAP2(9,12)* inhibits the metastasis of NPC cells in vivo

**Fig. 3.**
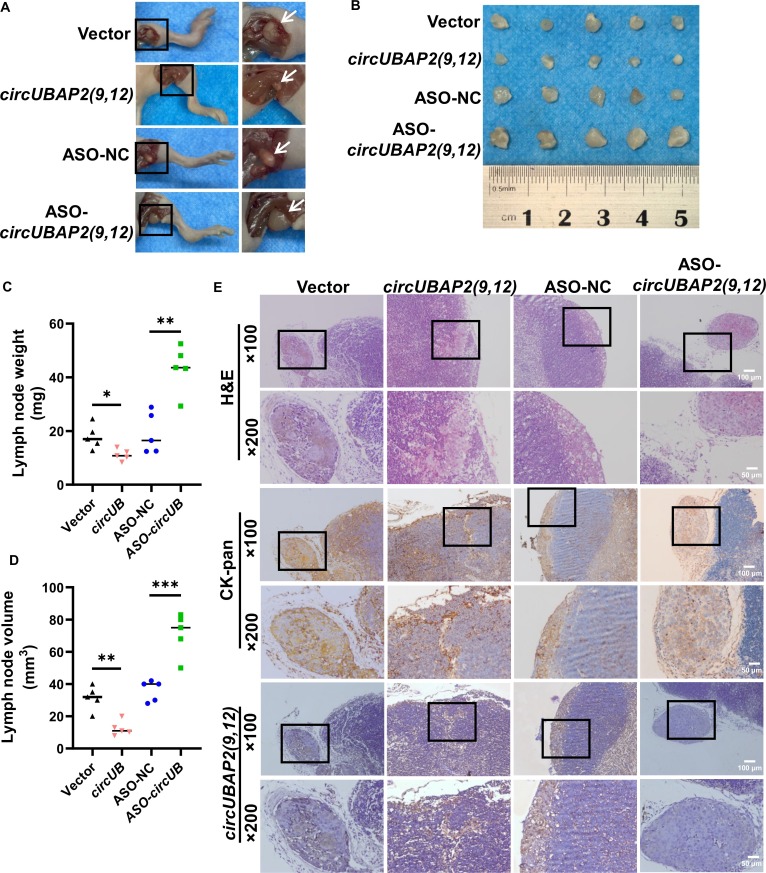
*circUBAP2(9,12)* inhibits the metastasis of NPC cells in vivo. (A) Representative images of inguinal lymph nodes from each experimental group. (B) Images showing the inguinal lymph nodes isolated from mice at 28 d after footpad inoculation with CNE2 cells (*n* = 5). (C and D) Quantification of lymph node weight and volume across the 4 groups. (E) Representative images of hematoxylin and eosin (H&E), immunohistochemical staining of CK-pan, and in situ hybridization for *circUBAP2(9,12)*. Magnification: ×100; scale bar = 100 μm. Magnification: ×200; scale bar = 50 μm. Results are shown as mean ± standard deviation from 5 separate biological repetitions. *P < 0.05; **P < 0.01; ***P < 0.001.

Over 80% of newly diagnosed NPC cases present with cervical lymph node involvement, and 6% to 8% exhibit concurrent distant metastasis [28]. To uncover the role of *circUBAP2(9,12)* in NPC metastasis, a xenograft model was established by injecting 2 × 10^6^ CNE2 cells (with *circUBAP2(9,12)* overexpression or knockdown) into the footpads of BALB/c nude mice. After 28 d, the inguinal lymph nodes were harvested. A marked difference was observed: both the weight and volume of metastatic lymph nodes were hugely reduced in the *circUBAP2(9,12)* overexpression group compared to those in the control, while the ASO-*circUBAP2(9,12)* group exhibited the opposite trend (Fig. 3A to D). Hematoxylin and eosin and cytokeratin-pan (CK-pan) staining verified that the extent of tumor cell infiltration in lymph nodes was markedly decreased in the overexpression group and increased in the knockdown group (Fig. 3E). ISH further revealed a stronger *circUBAP2(9,12)* signal in the *circUBAP2(9,12)* overexpression group and a weaker signal in the ASO-*circUBAP2(9,12)* group compared to that in the control (Fig. 3E). These findings suggest that *circUBAP2(9,12)* suppresses lymph node metastasis in NPC.

### *CircUBAP2(9,12)* suppresses EMT in NPC by destabilizing *ZEB2* mRNA

**Fig. 4.**
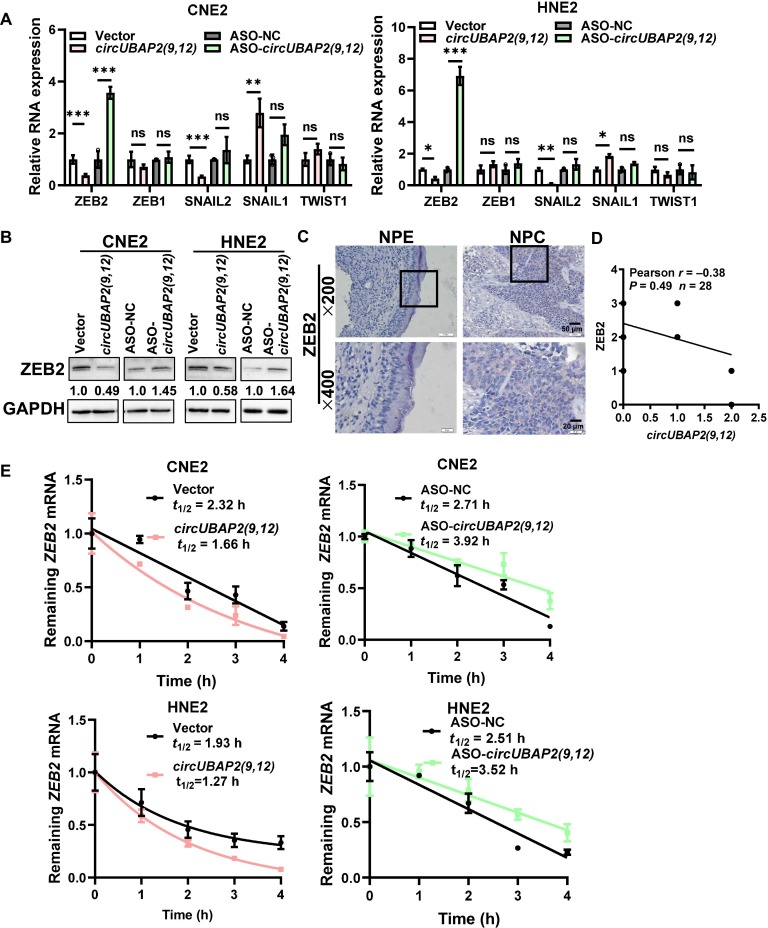
*circUBAP2(9,12)* suppresses epithelial–mesenchymal transition (EMT) in NPC by destabilizing zinc finger E-box binding homeobox 2 (*ZEB2*) mRNA. (A) qRT-PCR analysis of EMT-related transcription factors in NPC cells following *circUBAP2(9,12)* overexpression or knockdown. (B) Western blot analysis showing the effect of *circUBAP2(9,12)* on ZEB2 protein levels. (C) ZEB2 expression was evaluated by immunohistochemistry in 28 NPC tissue samples and 11 adjacent normal epithelial tissues. (D) Pearson correlation analysis revealed an inverse proportionality between *circUBAP2(9,12)* (in situ hybridization scores) and ZEB2 (immunohistochemistry scores) in 28 matched NPC tissue sections (serial sections from the same tumor blocks). (E) Measurement of *ZEB2* mRNA half-life was conducted by actinomycin D treatment followed by qRT-PCR in NPC cells. Results are shown as mean ± standard deviation from a minimum of 3 separate experimental repetitions. ns, not significant; *P < 0.05; **P < 0.01; ***P < 0.001. SNAIL1 and SNAIL2, snail family zinc finger 1 and 2; TWIST1, twist family BHLH transcription factor 1.

EMT [29] is a key cellular reprogramming process characterized by the loss of epithelial junctions—marked by reduced E-cadherin expression and the gain of mesenchymal markers (N-cadherin and vimentin [VIM]), enabling epithelial cells to acquire migratory and invasive properties [30,31]. To investigate the molecular mechanism by which *circUBAP2(9,12)* suppresses NPC cell migration and invasion, we examined several critical EMT-related transcription factors, including ZEB1, ZEB2, snail family zinc finger 1 (SNAIL1), SNAIL2, and twist family BHLH transcription factor 1 (TWIST1), all of which are known to repress E-cadherin transcription [30]. qRT-PCR analysis following *circUBAP2(9,12)* knockdown or overexpression revealed that only *ZEB2* mRNA was substantially down-regulated upon *circUBAP2(9,12)* overexpression (Fig. 4A), prompting us to focus on ZEB2 for further investigation. Western blot analysis confirmed that *circUBAP2(9,12)* negatively regulates ZEB2 at the protein level (Fig. 4B). Analysis of The Cancer Genome Atlas (TCGA) dataset showed that *ZEB2* is substantially elevated in head and neck squamous cell carcinoma (HNSC) (Fig. S2A), and microarray data from the GSE12452 dataset indicated similarly elevated expression in NPC tissues (Fig. S2B). To validate these findings, immunohistochemistry (IHC) was performed on NPC tissue samples and adjacent normal epithelial tissues, revealing markedly higher ZEB2 expression in NPC tissues (Fig. 4C).

Kaplan–Meier survival curves indicate notably poorer overall survival for NPC patients with high ZEB2 expression relative to those with low expression (Fig. S2C). Furthermore, Pearson correlation analysis of the same 28 tumor samples revealed an inverse relationship between the ISH scores of *circUBAP2(9,12)* and IHC scores of ZEB2 (Fig. 4D).

Numerous studies have shown that circRNAs can competitively bind miRNAs by acting as competitive endogenous RNAs (ceRNAs), to modulate target mRNA levels [32]. However, this mechanism typically results in a positive correlation between the circRNA and its target mRNA, which contradicts our observations. Since *circUBAP2(9,12)* negatively regulates *ZEB2* mRNA levels, we hypothesized that it may affect *ZEB2* mRNA stability rather than acting through miRNA sponging. To test this, we treated NPC cells overexpressing or silenced for *circUBAP2(9,12)* with actinomycin D and assessed *ZEB2* mRNA decay. The results revealed that overexpression of *circUBAP2(9,12)* markedly reduced the stability of *ZEB2* mRNA (Fig. 4E). These findings suggest that *circUBAP2(9,12)* negatively regulates *ZEB2* expression by promoting mRNA degradation.

### *CircUBAP2(9,12)* suppresses *ZEB2* expression through competitive binding to AUF1

Extensive research has demonstrated that mRNA stability is largely regulated by the 3′UTR. To determine whether additional proteins are involved in *circUBAP2(9,12)*-mediated modulation of *ZEB2* mRNA stability, we analyzed the 3′UTR sequence of *ZEB2* and found an enrichment of AU nucleotides (Fig. S2D), indicative of potential AREs [33]. AUF1 (encoded by the heterogeneous nuclear ribonucleoprotein D [*HNRNPD*] gene) is one of the most extensively studied ARE-binding proteins and can modulate mRNA stability by forming ribonucleoprotein (RNP) complexes [34]. We therefore hypothesized that *circUBAP2(9,12)* might suppress *ZEB2* mRNA stability by interacting with AUF1.

**Fig. 5.**
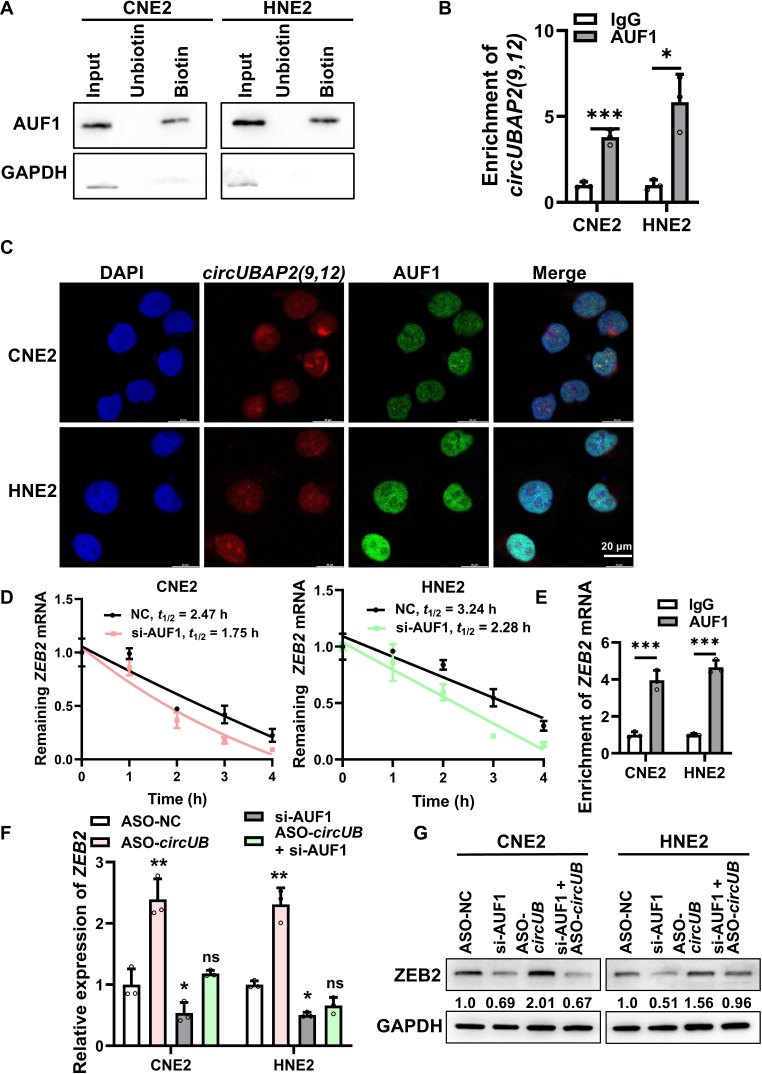
*circUBAP2(9,12)* suppresses *ZEB2* expression through competitive binding to adenylate-uridylate-rich binding factor 1 (AUF1). (A) RNA pulldown assay demonstrating the interaction between *circUBAP2(9,12)* and AUF1. (B) Ribonucleoprotein immunoprecipitation assay using anti-AUF1 antibody to detect the enrichment of *circUBAP2(9,12)*. (C) Co-localization of *circUBAP2(9,12)* (red) and AUF1 (green) in NPC cells as visualized by fluorescence in situ hybridization. Scale bar = 20 μm. (D) *ZEB2* mRNA half-life following AUF1 knockdown and actinomycin D treatment was detected using qRT-PCR. (E) Ribonucleoprotein immunoprecipitation assay with anti-AUF1 antibody confirming the binding of AUF1 to *ZEB2* mRNA. (F) qRT-PCR quantification of *ZEB2* mRNA levels after single or combined knockdown of AUF1 and *circUBAP2(9,12)*. (G) ZEB2 protein levels following individual or simultaneous knockdown of AUF1 and *circUBAP2(9,12)* were measured using western blot. Results are shown as mean ± standard deviation from a minimum of 3 separate experimental repetitions. ns, not significant; *P < 0.05; **P < 0.01; ***P < 0.001. IgG, immunoglobulin G; si-AUF1, AUF1 siRNA.

To test this, RNA pulldown and ribonucleoprotein immunoprecipitation (RIP) assays confirmed a direct interaction between *circUBAP2(9,12)* and AUF1 (Fig. 5A and B). Fluorescence ISH showed nuclear co-localization of *circUBAP2(9,12)* and AUF1 (Fig. 5C). Analysis of the TCGA-HNSC cohort revealed that *HNRNPD* is significantly up-regulated in 519 tumor tissues versus 44 normal samples (Fig. S3A), and similar overexpression was observed in NPC tissues in the GSE12452 dataset (31 NPC vs. 10 normal tissues) (Fig. S3B). Western blot showed that neither overexpression nor knockdown of *circUBAP2(9,12)* affected AUF1 protein levels (Fig. S3C). Conversely, AUF1 knockdown via small interfering RNA (siRNA) did not alter *circUBAP2(9,12)* expression (Fig. S3D to F), indicating no reciprocal regulation.

To delineate the functional role of AUF1 in regulating *ZEB2* mRNA, we assessed *ZEB2* mRNA half-life following AUF1 knockdown and actinomycin D treatment. The results showed reduced *ZEB2* mRNA stability upon AUF1 silencing (Fig. 5D). RIP assays also confirmed AUF1 binding to *ZEB2* mRNA (Fig. 5E). To evaluate whether *circUBAP2(9,12)* modulates ZEB2 expression through AUF1, we performed single and combined knockdown of *circUBAP2(9,12)* and AUF1 in NPC cells. qRT-PCR and western blot analyses suggested that co-silencing AUF1 partially reversed the increase of ZEB2 expression prompted by *circUBAP2(9,12)* knockdown (Fig. 5F and G). Collectively, these results suggest that *circUBAP2(9,12)* negatively regulates *ZEB2* mRNA stability through its interaction with AUF1.

### *CircUBAP2(9,12)* sequesters AUF1 and attenuates its binding to *ZEB2* mRNA

**Fig. 6.**
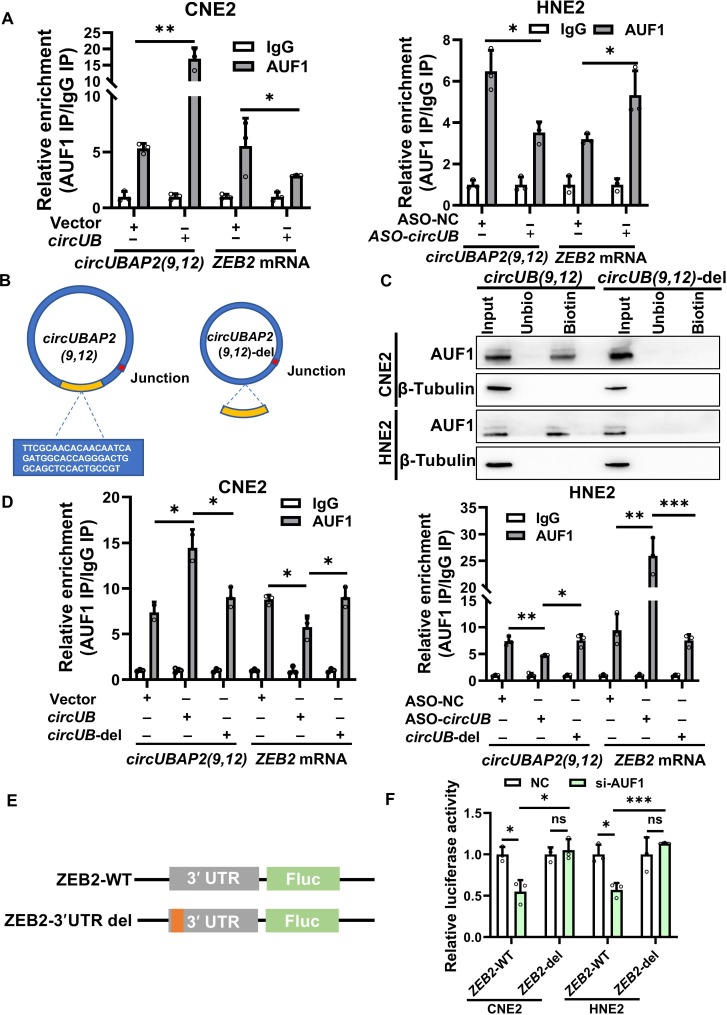
*circUBAP2(9,12)* sequesters AUF1 and attenuates its binding to *ZEB2* mRNA. (A) Ribonucleoprotein immunoprecipitation assay showing AUF1 binding to *ZEB2* mRNA or*circUBAP2(9,12)* following *circUBAP2(9,12)* overexpression or knockdown. (B) Schematic representation of the full-length *circUBAP2(9,12)* construct and the truncated mutant *circUBAP2(9,12)*-del lacking the 301- to 352-nt region. (C) RNA pulldown assay assessing the binding affinity of AUF1 to wild-type *circUBAP2(9,12)* versus *circUBAP2(9,12)*-del. (D) Ribonucleoprotein immunoprecipitation assay evaluating the effect of *circUBAP2(9,12)* or *circUBAP2(9,12)*-del on AUF1 binding to *ZEB2* mRNA*circUBAP2(9,12)*. (E) Schematic diagram of luciferase reporter constructs inserting either the wild-type *ZEB2* 3′ untranslated region (3′UTR) or a truncated version lacking nucleotides 481 to 578. (F) Dual-luciferase reporter assay assessing the function of AUF1 knockdown on luciferase activity driven by ZEB2-WT or ZEB2-3′UTR-del constructs. Results are shown as mean ± standard deviation from a minimum of 3 separate experimental repetitions. ns, not significant; *P < 0.05; **P < 0.01; ***P < 0.001. IP, immunoprecipitation; Unbio, unbiotin; WT, wild type.

To further investigate how *circUBAP2(9,12)* interacts with AUF1 and regulates *ZEB2* mRNA stability, we used RNAhybrid [35,36] to predict potential interaction between AUF1 and *circUBAP2(9,12)* or *ZEB2* mRNA. The analysis revealed that the 301- to 352-nt region of *circUBAP2(9,12)* and the 481- to 578-nt region of *ZEB2* mRNA both potentially interact with the same domain of AUF1 (Fig. S4A). Based on this, we hypothesized that *circUBAP2(9,12)* competes with *ZEB2* mRNA for AUF1 binding, thereby reducing AUF1-mediated stabilization of *ZEB2* mRNA. RIP assays supported this hypothesis: overexpression of *circUBAP2(9,12)* reduced AUF1 binding to *ZEB2* mRNA while increasing AUF1 association with *circUBAP2(9,12)*; conversely, silencing *circUBAP2(9,12)* enhanced AUF1 binding to *ZEB2* mRNA and reduced its interaction with *circUBAP2(9,12)* (Fig. 6A). These findings suggest a competitive binding mechanism. To validate the critical binding region, we constructed a mutant version of *circUBAP2(9,12)* lacking the predicted AUF1-binding site (301 to 352 nt), designated *circUBAP2(9,12)*-del (Fig. 6B), and transfected it or the wild-type construct into CNE2 and HNE2 cells. RNA pulldown assays showed that AUF1 bound robustly to wild-type *circUBAP2(9,12)*, while its interaction with *circUBAP2(9,12)*-del was nearly abolished (Fig. 6C). We further performed RIP assays following transfection of either construct. AUF1 enrichment was observed only with overexpression of wild-type *circUBAP2(9,12)*, but not with the mutant, which showed binding levels comparable to those in the vector control. Similarly, AUF1 binding to *ZEB2* mRNA was reduced only in the wild-type group, not in the *circUBAP2(9,12)*-del group. In contrast, *circUBAP2(9,12)* knockdown increased AUF1 binding to *ZEB2* mRNA, but this effect was absent with the *circUBAP2(9,12)*-del mutant (Fig. 6D). Finally, to assess the functional impact, we examined cell migration, invasion, and *ZEB2* mRNA stability following overexpression of wild-type or mutant *circUBAP2(9,12)*. The mutant construct failed to suppress migration and invasion or stabilize *ZEB2* mRNA, in contrast to the wild type (Fig. S4B to D). These data confirm that *circUBAP2(9,12)* binds AUF1 via the 301- to 352-nt region and competitively interferes with AUF1-mediated stabilization of *ZEB2* mRNA.

To further confirm that the *circUBAP2(9,12)*–AUF1 complex regulates *ZEB2* mRNA stability via the AUF1-binding site within the *ZEB2* 3′UTR, we constructed luciferase reporter plasmids using the pMIR-REPORT vector. One construct contained the full-length wild-type 3′UTR of ZEB2 (ZEB2-WT), while the other contained a truncated version lacking the AUF1-binding site (deletion of nucleotides 481 to 578), referred to as ZEB2-3′UTR-del (Fig. 6E). Dual-luciferase reporter assays demonstrated that co-transfection of AUF1 siRNA (si-AUF1) with the ZEB2-WT reporter markedly reduced luciferase activity, indicating that AUF1 stabilizes *ZEB2* mRNA in a 3′UTR-dependent manner. In contrast, co-transfection of si-AUF1 with the ZEB2-3′UTR-del construct had no interference on luciferase luminance (Fig. 6F), confirming that AUF1 binds specifically to the 481- to 578-nt region of the *ZEB2* 3′UTR. Collectively, these findings substantiate a model in which the 301- to 352-nt region of *circUBAP2(9,12)* is required for AUF1 binding and competitively inhibits AUF1 interaction with the 481- to 578-nt region of the *ZEB2* 3′UTR, thereby reducing *ZEB2* mRNA stability.

### *CircUBAP2(9,12)* inhibits the migration and invasion of NPC via the AUF1–ZEB2 axis

**Fig. 7.**
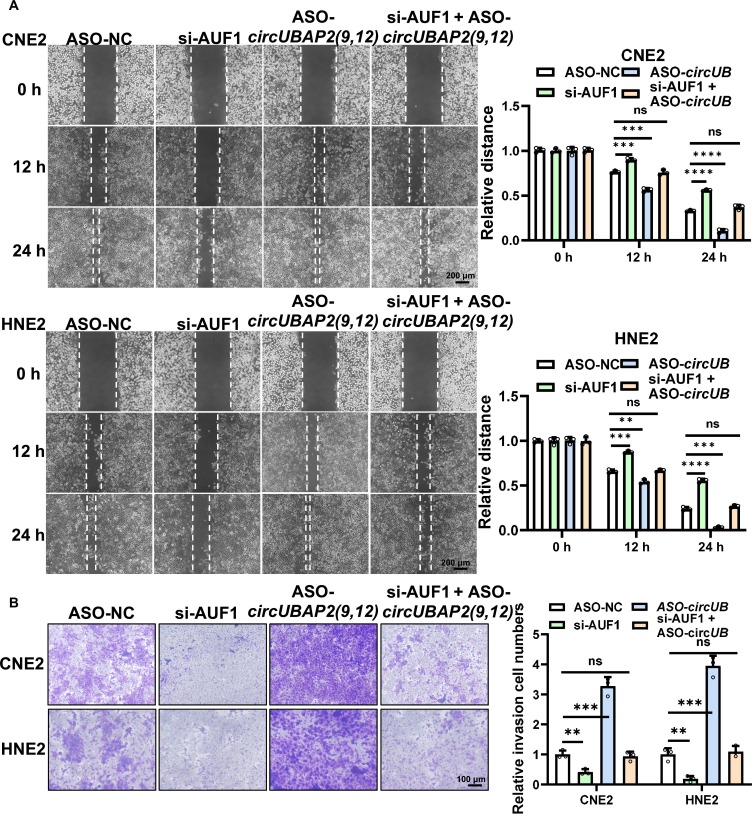
*circUBAP2(9,12)* attenuates the migration and invasion of NPC via AUF1. (A) NPC cell migration was tested by wound healing assays following knockdown of *circUBAP2(9,12)*, AUF1, or both simultaneously. The scratch width was quantified using ImageJ. Scale bar = 200 μm. (B) NPC cell invasion was measured by transwell assays under the same knockdown conditions. Quantification of invading cells was done using ImageJ. Scale bar = 100 μm. All experiments were conducted in triplicate (biological replicates). Results are shown as mean ± standard deviation from a minimum of 3 separate biological repetitions. ns, not significant; **P < 0.01; ***P < 0.001; ****P < 0.0001.

**Fig. 8.**
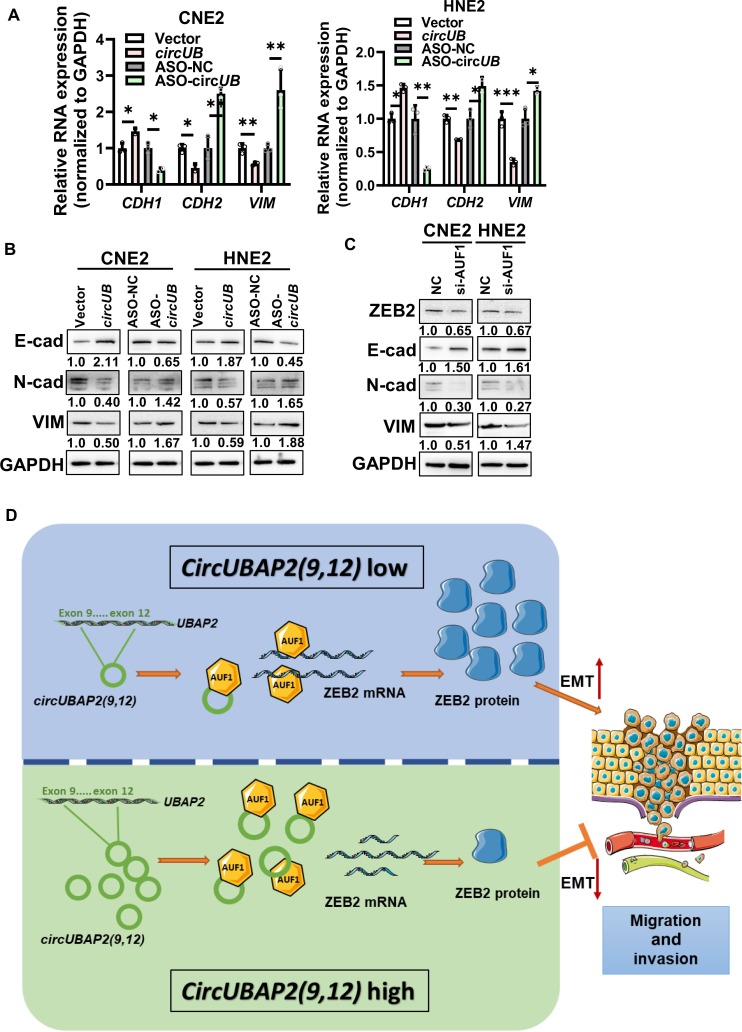
*circUBAP2(9,12)* inhibits EMT in NPC. (A) qRT-PCR analysis of cadherin-1 (*CDH1*), *CDH2*, and vimentin (*VIM*) mRNA levels following *circUBAP2(9,12)* knockdown or overexpression*.* (B) Western blot analysis of E-cadherin, N-cadherin, and VIM protein expression after *circUBAP2(9,12)* knockdown or overexpression. (C) Western blot analysis of ZEB2, and EMT marker expression following AUF1 knockdown. (D) Schematic working model: Low *circUBAP2(9,12)* expression allows AUF1 to bind *ZEB2* mRNA, enhancing its stability and promoting NPC cell migration and invasion. High *circUBAP2(9,12)* expression sequesters AUF1, preventing its interaction with *ZEB2* mRNA, thereby reducing *ZEB2* mRNA stability and suppressing migratory and invasive potential. Results are shown as mean ± standard deviation from a minimum of 3 separate technical repetitions. *P < 0.05; **P < 0.01; ***P < 0.001. E-cad, E-cadherin; N-cad, N-cadherin.

To investigate whether *circUBAP2(9,12)* competitively binds to AUF1 and thereby modulates the migratory and invasive behavior of NPC cells, we performed functional rescue assays following individual or combined knockdown of *circUBAP2(9,12)* and AUF1. Functional rescue experiments revealed an antagonistic relationship: silencing AUF1 suppressed NPC cell migration and invasion, while *circUBAP2(9,12)* knockdown enhanced these phenotypes. Co-silencing both partially restored the migratory and invasive capacities, suggesting a competitive functional interplay between *circUBAP2(9,12)* and AUF1 (Fig. 7A and B). ZEB2 is a key EMT-related transcription factor known to repress E-cadherin (encoded by cadherin-1 [*CDH1*]) and promote the expression of N-cadherin (encoded by *CDH2*) and VIM [37–39]. To explore the involvement of *circUBAP2(9,12)* in EMT regulation, we examined EMT marker expression. qRT-PCR and western blot showed that ASO-*circUBAP2(9,12)* down-regulated E-cadherin and up-regulated N-cadherin and VIM (Fig. 8A and B). Conversely, AUF1 knockdown reduced the expression of ZEB2, N-cadherin, and VIM while increasing E-cadherin levels (Fig. 8C). To further validate that *circUBAP2(9,12)* exerts its effects through ZEB2-mediated EMT, rescue experiments were conducted. Silencing ZEB2 partially reversed the ASO-*circUBAP2(9,12)*-induced migration and invasion enhancement, supporting the role of ZEB2 as a downstream effector of *circUBAP2(9,12)* (Fig. S5A and B).

Additionally, western blot analysis showed that co-transfection of *circUBAP2(9,12)* and ZEB2 partially reversed the suppressive effect of *circUBAP2(9,12)* on N-cadherin and VIM expression. Conversely, co-transfection of ASO-*circUBAP2(9,12)* with si-ZEB2 partially rescued E-cadherin expression, which was otherwise reduced by ASO-*circUBAP2(9,12)* alone (Fig. S5C and D). Together, these findings indicate that *circUBAP2(9,12)* restricts NPC cell migration and invasion by interfering with AUF1–ZEB2 interaction.

## Discussion

NPC arises from the nasopharyngeal epithelium and exhibits a distinct geographical distribution [40]. Approximately 80% of newly diagnosed NPC cases present with cervical lymph node involvement [41] and 30% to 40% developing distant metastases following treatment [42]. Understanding the molecular mechanisms underlying NPC progression is therefore critical [43]. In this study, we identify *circUBAP2(9,12)* as a novel anti-tumor circRNA and reveal the *circUBAP2(9,12)*–AUF1–ZEB2 axis as a potential therapeutic vulnerability that may help address the challenge of NPC metastasis.

*UBAP2*, which encodes a protein containing a ubiquitin-associated domain, serves as a critical regulator in the ubiquitination pathway and has been implicated in the progression of various malignant tumors [44]. Comprising 32 exons, *UBAP2* gives rise to numerous circRNA isoforms, some of which have been previously reported. For example, *hsa_circ_0001846*, generated by the circularization of exons 11 to 14 of *UBAP2* pre-mRNA, has been shown to sequester miR-143, thereby up-regulating Bcl-2 and promoting osteosarcoma progression [45]. Another isoform, *hsa_circ_0003945*, formed by the circularization of exons 11 and 12, functions as a ceRNA for miR-194-3p, enhancing MMP9-mediated oncogenic activity in hepatocellular carcinoma [46]. In this study, we identified *circUBAP2(9,12)* (*hsa_circ_0001847*), a novel circRNA derived from the back-splicing of exons 9 to 12 of *UBAP2*, which has not been previously reported. Notably, *circUBAP2(9,12)* displayed the highest expression abundance among all *UBAP2*-derived circRNAs in NPC tissues (even surpassing some known oncogenic *circUBAP2* isoforms; Table S7), highlighting the tissue-specific nature of circRNA expression. Importantly, *circUBAP2(9,12)* was substantially lower in clinical NPC samples and was found to suppress the migration and invasion of NPC cells.

EMT in malignant tumors is associated with the acquisition of various traits, including resistance to apoptosis, therapy-induced senescence, altered cell death and DNA repair mechanisms, and enhanced stem-cell-like properties—all of which contribute to tumor metastasis [47]. EMT is orchestrated by several key transcription factors, such as SNAIL1, SNAIL2, TWIST, and the ZEB family, which function as molecular switches driving the transition [48]. Given that *circUBAP2(9,12)* is predominantly localized in the nucleus and EMT-related transcription factors regulate the expression of epithelial and mesenchymal markers, we first examined its effect on EMT transcription factors. Our results revealed that *circUBAP2(9,12)* specifically inhibits the expression and mRNA stability of *ZEB2*. As a central regulator of EMT, ZEB2 is strongly associated with poor clinical prognosis and tumor metastasis and is frequently co-expressed with core mesenchymal markers in various malignancies [49]. Although the role of ZEB2 in NPC has been studied in the context of miRNA-mediated regulation, circRNA-based modulation of ZEB2 has not previously been reported. This study provides new insights by demonstrating that *circUBAP2(9,12)* inhibits NPC progression through the suppression of *ZEB2* mRNA stability, underscoring its functional significance in EMT and metastasis.

Numerous studies have demonstrated that circRNAs exert multimodal regulatory functions through various mechanisms [50], including regulation of their parental genes, formation of circRNA–protein complexes, acting as miRNA sponges, and, in some cases, translation into peptides. In our study, we found that neither silencing nor overexpressing *circUBAP2(9,12)* affected the expression of its parental gene. Moreover, in silico predictions suggest that *circUBAP2(9,12)* lacks coding potential and is unlikely to encode small peptides. While circRNAs function as ceRNAs by sponging miRNAs, this typically results in a positive correlation between circRNA and mRNA. However, we observed that *circUBAP2(9,12)* negatively regulates *ZEB2* mRNA levels, suggesting that it does not act through a miRNA sponging mechanism in this context. Recent advances have highlighted the importance of circRNA–protein interactions in modulating protein expression, localization, and function. Based on this, we hypothesized that *circUBAP2(9,12)* may exert its regulatory effects by binding to specific RBPs. Analysis of the 3′UTR sequence of *ZEB2* mRNA revealed a high density of AREs, suggesting a potential interaction with AUF1, a well-characterized ARE-binding protein known to influence mRNA stability.

AUF1, a member of the widely expressed heterogeneous nuclear RNP family, binds with high affinity to AREs typically located within the 3′UTRs of mRNAs, playing a key role in regulating mRNA stability [51]. Depending on the cellular context and target transcript, AUF1 can either promote or reduce mRNA stability. For instance, circ-DNMT1 enhances the nuclear localization of AUF1, thereby reducing the degradation of *DNMT1* mRNA and promoting its translation, which contributes to breast cancer progression [52]. On the contrary, *circUBE3A(2,3,4,5)* directly interacts with AUF1, facilitating its nuclear import, resulting in decreased cytoplasmic AUF1, destabilization of *MTHFD2* mRNA, and suppression of prostate cancer cell migration and invasion [24]. In the present study, we demonstrate that AUF1 enhances the stability of *ZEB2* mRNA in NPC. Furthermore, *circUBAP2(9,12)* was found to competitively bind to the same region of AUF1 as *ZEB2* mRNA, thereby preventing AUF1 from interacting with *ZEB2*. This competitive binding reduces *ZEB2* mRNA stability and subsequently inhibits the migration and invasion of NPC cells.

In summary, we identified a novel circRNA, *circUBAP2(9,12)*—generated from exons 9 to 12 of *UBAP2*—which exhibits reduced expression in NPC tissues and functions to restrict tumor cell migration and invasion. Clinically, low *circUBAP2(9,12)* expression is linked to unfavorable clinical outcomes in NPC. Mechanistically, reduced *circUBAP2(9,12)* expression in tumor tissues limits its ability to sequester AUF1, thereby allowing increased AUF1 binding to *ZEB2* mRNA, which stabilizes *ZEB2* transcripts and promotes EMT, migration, and invasion. Conversely, in normal tissues where *circUBAP2(9,12)* is highly expressed, it competitively binds to AUF1, reducing AUF1’s interaction with *ZEB2* mRNA, leading to destabilization of *ZEB2* mRNA and suppression of migration and invasion (Fig. 8D). These findings uncover a previously unreported role of *circUBAP2(9,12)* in regulating NPC metastasis and highlight the *circUBAP2(9,12)*–AUF1–ZEB2 axis as a promising therapeutic target in NPC management.

## Materials and Methods

### Cell culture and transfection

NPC cell lines HNE2, CNE2, and HK1 were purchased from the Cell Center of Central South University. Cells cultures were established using RPMI 1640 medium (Gibco, USA) enriched with 10% heat-inactivated fetal bovine serum (FBS; Gibco, USA). All cultures were maintained under standard mammalian cell conditions (37 °C, 5% CO_2_, >90% humidity). Pre-experiment *Mycoplasma* testing (PCR) yielded negative results for all cell lines.

The full-length *circUBAP2(9,12)* (*hsa_circ_0001847*) was PCR-amplified and directionally ligated into the pcDNA3.1(+) circRNA Mini Vector [53], generously provided by Dr Yong Li (Baylor College of Medicine). The *ZEB2* overexpression construct was purchased from Tsingke Biotechnology Co., Ltd. (Changsha, China). All recombinant plasmids were validated by bidirectional Sanger sequencing (Tsingke Biotechnology Co., Ltd., Changsha, China). ASOs targeting the *circUBAP2(9,12)* junctional region and AUF1- or ZEB2-specific siRNAs were designed and synthesized by RiboBio (Guangzhou, China). Transfection was performed with 50 nM ASO-*circUBAP2(9,12)* or siRNA complexed with HiPerFect reagent (QIAGEN, Germany) at a 1:1 ratio in serum-free medium, while plasmid transfection was performed with Neofect at a 1:2 ratio (Neofect Biotech Co., Ltd., China). The sequences of ASO-*circUBAP2(9,12)*, si-AUF1, and si-ZEB2 are provided in Table S1.

### Clinical samples

Human tissue specimens were obtained from 2 independent clinical cohorts. The first cohort comprised 34 treatment-naive NPC biopsies and 8 nasopharyngitis samples (Table S2) for detecting the expression of *circUBAP2(9,12)* by qRT-PCR. The second cohort included 76 primary NPC tumors paired with 48 histologically normal surgical margins (Table S3) for spatial expression profiling. All specimens were collected at Hunan Cancer Hospital following standardized protocols and with approval from the Ethics Committee of Central South University. Histopathological diagnoses were independently confirmed by 2 blinded senior pathologists. All participants provided written informed consent prior to study enrollment.

### IHC and ISH

IHC staining for CK-pan and ZEB2 was performed using a standardized protocol with IHC Detection Kit (MXB Biotechnologies, Fuzhou, China). Primary antibody incubations were performed for 16 h at 4 °C in blocking buffer at optimized concentrations: CK-pan (1:200) and ZEB2 (1:150). Detection of *circUBAP2(9,12)* was carried out by ISH using digoxigenin-labeled probes (sequences listed in Table S4) from Sangon Biotech (Shanghai, China), in conjunction with Enhanced Sensitive ISH Detection Kit (MK1030, BOSTER, China). A dual-parameter semiquantitative scoring system was applied: staining intensity was graded as 0 (negative), 1 (pale yellow), 2 (light brown), or 3 (dark brown); the percentage of positive area relative to total tissue area was quantified as 0 (<25%), 1 (25% to 50%), 2 (51% to 75%), or 3 (>75%) [54]. The final expression score was calculated by multiplying the staining intensity score by the percentage of positive cells, with a total score ≥5 indicating high expression and <5 indicating low expression. All assessments were independently performed by 2 blinded pathologists, with an inter-rater reliability *κ* value greater than 0.8.

### RNA isolation, reverse transcription, and qRT-PCR

TRIzol reagent was used for total RNA extraction (Life Technologies, USA). Reverse transcription was done using the HiScript II Q RT SuperMix for qPCR kit (Vazyme, China). qRT-PCR was conducted on CFX Real-Time PCR System (Bio-Rad, USA) using 2× SYBR Green qPCR Master Mix (Bimake, USA). Relative expression levels were calculated using the 2^−ΔΔCt^ method with glyceraldehyde-3-phosphate dehydrogenase as (GAPDH) the endogenous control. All primers were synthesized by Tsingke Biotechnology Co., Ltd., and their sequences are listed in Table S5.

### RNase R digestion and actinomycin D treatment

Total RNA extracted from CNE2 and HNE2 cells was incubated with RNase R (1 U/μg; Epicentre, USA) at 37 °C for 15 min. Following treatment, RNA was extracted with TRIzol; the expression levels of *circUBAP2(9,12)* and linear *UBAP2* mRNA were quantified by qRT-PCR.

Forty-eight hours after transfection, NPC cells were treated with actinomycin D (Sigma-Aldrich, USA) or dimethyl sulfoxide at a final concentration of 1 μg/ml and harvested at indicated time points to evaluate RNA stability. Total RNA was extracted with TRIzol; an equal volume of RNA was reverse-transcribed into complementary DNA followed by qRT-PCR to assess mRNA decay kinetics. Remaining RNA levels at different time points were normalized to the level at 0 h. Nonlinear regression under exponential-one phase decay mode (GraphPad Prism) was used to assess mRNA half-life.

### Cytosolic/nuclear fractionation assay

Subcellular fractionation of NPC cells was performed using Cytoplasmic & Nuclear RNA Purification Kit (Invitrogen, USA). Briefly, cells were lysed in a hypotonic buffer, followed by differential centrifugation to isolate cytoplasmic and nuclear fractions, followed by RNA extraction from each compartment.

### Wound healing assay

Once transfected cells reached full confluence, a uniform scratch was introduced using a 10-μl pipette tip. To minimize variability, care was taken to maintain a consistent scratch width. The medium was then substituted with RPMI 1640 supplemented with 2% FBS and 1.8 mmol/l hydroxyurea (Sigma-Aldrich, USA) to inhibit cell proliferation. Cell migration was assessed by capturing bright-field images at 0, 12, and 24 h post-scratch on an inverted microscope. Relative wound width was calculated using ImageJ by measuring 3 equidistant points along each scratch and normalizing to the 0-h baseline.

### Transwell assay

Twenty-four hours after transfection, Matrigel (BD Biosciences, San Jose, CA) was diluted 1:9 with RPMI 1640 medium on ice. A 20-μl aliquot of diluted Matrigel was uniformly coated onto the upper chamber of 8-μm-pore transwell inserts (8-μm pore size; Corning, NY) and incubated at 37 °C for 2 h to allow gel solidification. Subsequently, 20,000 transfected NPC cells were carefully seeded into the Matrigel-coated upper chamber, while the lower chamber contained RPMI 1640 medium with 20% FBS. After incubation at 37 °C for 24 to 48 h, cells remaining on the upper surface were gently removed with a cotton swab. The invasive cells on the lower surface were fixed with 4% paraformaldehyde, stained with 0.1% crystal violet, and imaged using an inverted microscope. Cell numbers were quantified using the ImageJ software.

### RIP assay

RNA immunoprecipitation was performed using a RIP kit (Millipore) per the manufacturer’s instructions. Briefly, magnetic beads and specific antibodies were incubated with cell lysates, followed by RNA isolation from the immunoprecipitates and subsequent analysis by qRT-PCR, as described in previous studies [5,55].

### RNA pulldown

*CircUBAP2(9,12)*-biotin probes were transfected into NPC cells; 48 h after transfection, cells were lysed using RIP buffer (25 mM Tris–HCl, 0.5 mM dithiothreitol, 150 mM KCl, and 0.5% NP-40) [42]. The lysates were then incubated with 50 μl of streptavidin Dynabeads (Invitrogen, USA) and rotated for 16 h at 4 °C. The bead-bound complexes were washed 6 times with RIP buffer, each wash involving 5 min of rotation. Subsequently, western blot was performed to assess the expression of associated proteins. The sequences of the biotin-labeled probes are provided in Table S4.

### Western blot

Proteins were extracted from NPC cells employing radioimmunoprecipitation assay buffer (Beyotime Biotechnology, China) supplemented with a protease inhibitor cocktail (Roche Applied Sciences, Germany). Protein concentrations were quantified by the bicinchoninic acid assay (Bio-Rad, USA). Denatured protein samples (30 to 50 μg) were separated on 10% sodium dodecyl sulfate–polyacrylamide gel electrophoresis gels. Following electrophoresis, proteins were transferred onto 0.2-μm polyvinylidene difluoride membranes (Millipore) by wet transfer. Membranes were blocked with 5% skimmed milk in phosphate-buffered saline with Tween 20 (PBST) for 1 h at room temperature and then incubated overnight at 4 °C with primary antibodies. After washing 3 times for 10 min each with PBST, membranes were incubated with secondary antibodies for 2 h at room temperature. Immunoreactive bands were visualized using Millipore ECL substrate and quantified with ImageJ. Antibody details are listed in Table S6.

### Luciferase reporter assay

For the luciferase reporter assay, CNE2 or HNE2 cells were co-transfected with luciferase reporter vectors containing either wild-type ZEB2 3′UTR (ZEB2-WT) or a 3′UTR deletion mutant (ZEB2 3′UTR-del), along with the pRL-TK *Renilla* vector (Promega, USA). Forty-eight hours after transfection, luciferase activity was measured using Dual-Luciferase Reporter Assay System (Promega, USA). Relative luminescence units were derived through normalization of firefly luciferase readings against internal *Renilla* luciferase controls, thereby assessing *ZEB2* 3′UTR regulatory activity.

### Fluorescence ISH

To evaluate the subcellular localization of *circUBAP2(9,12)* and its co-localization with AUF1, a digoxin-labeled probe targeting the junction region of *circUBAP2(9,12)* was synthesized by GenePharma (Shanghai, China). Imaging was performed using a confocal laser scanning microscope (Leica, Germany). Probe sequences are listed in Table S4.

### Animal experiments

Female BALB/c nude mice (5 to 6 weeks old) were divided into 4 groups through random allocation (*n* = 5 per group). To establish a lymph node metastasis model, CNE2 cells (2 × 10^6^) transfected with either negative control ASO (ASO-NC), ASO-*circUBAP2(9,12)*, empty vector, or *circUBAP2(9,12)* overexpression plasmid were injected into the footpad. Following a 28-d observation period, animals were humanely sacrificed with subsequent collection of tumor-draining (ipsilateral inguinal) lymph nodes for hematoxylin and eosin staining, *circUBAP2(9,12)* ISH, CK-pan immunostaining, and metastasis assessment. All animal protocols were approved by the Animal Welfare Ethics Committee of Central South University (Changsha, China) (Protocol CSU-2024-0333).

### Statistical analysis

All results were confirmed in triplicate biological experiments. Continuous data are expressed as mean ± standard deviation. Comparisons between 2 groups were performed using the Student *t* test, while one-way analysis of variance was applied for multiple group comparisons. Statistical significance was defined as *P* < 0.05, *P* < 0.01, *P* < 0.001, and *P* < 0.0001. Data visualization and statistical analyses were carried out using GraphPad Prism version 9.0 (GraphPad Software, San Diego, CA).

## Ethical Approval

This study was approved by the Ethics Committee of Central South University. Written informed consent was acquired from each participant.

## Data Availability

The datasets generated during this study are available from the corresponding authors on reasonable request.
