# Supplementary material for: CircUBAP2(9,12) Inhibits Nasopharyngeal Carcinoma Invasion and Metastasis by Down-regulating ZEB2 through Competitive Binding to AUF1
Source: Research (Wash D C). 2025 Nov 4;8:0936. doi: 10.34133/research.0936 (PMC12583797; doi:10.34133/research.0936)
Supplement: Supplementary 1 — Figs. S1 to S5 Tables S1 to S7 [file research.0936.f1.zip › Figs S1-S5.pdf]

Fig S1

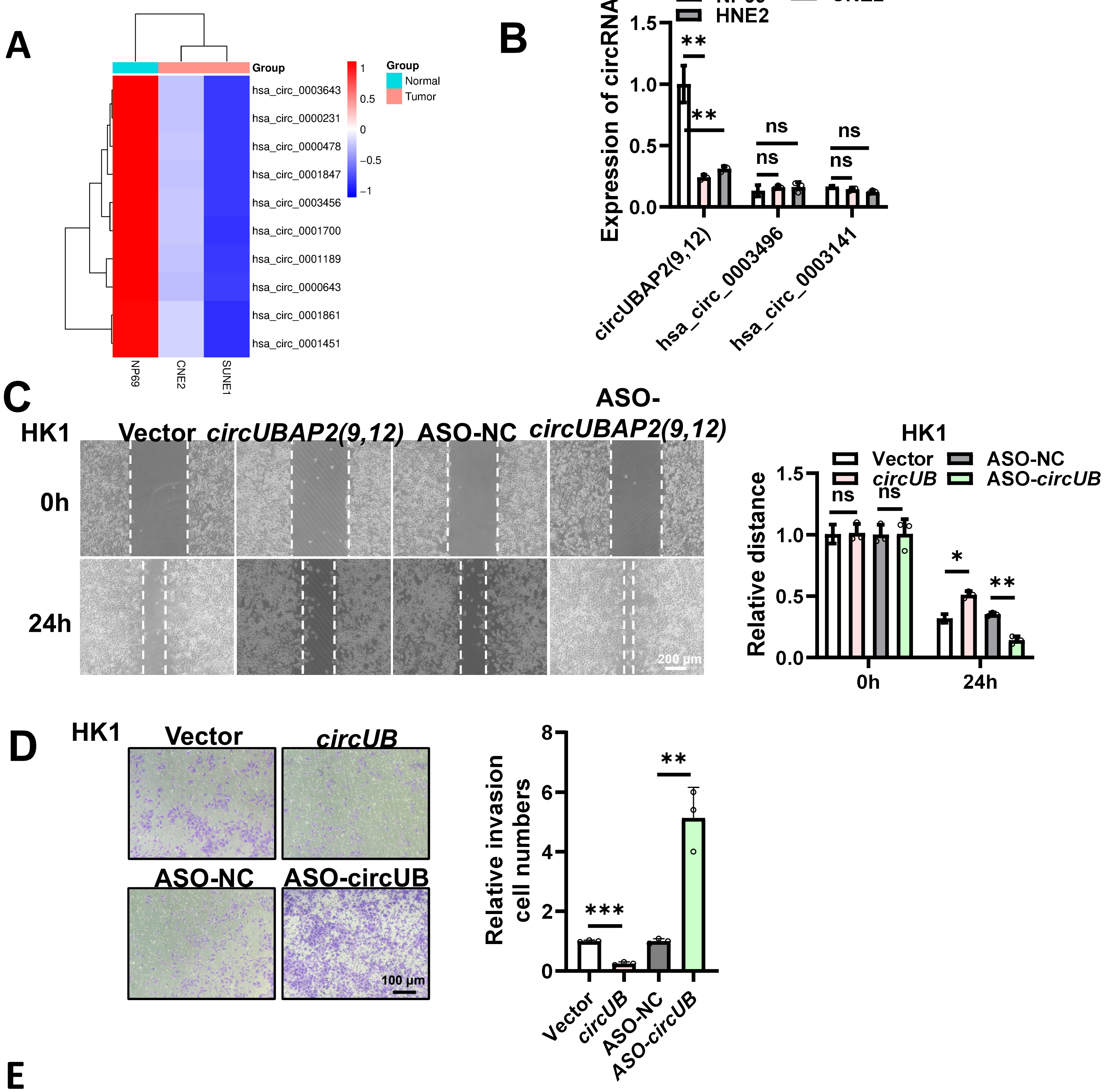

E

Protein coding potential

	Parameter Index		
IRES Elements	Position (start--end)	R Score	With Pseudoknot (Y/N)
	271--377	1.486197	Y
	192--323	1.318095	N
Open Reading Frame (ORF)	No open reading frame was found ! (Protein length less than 100aa)		
Protein Features	The possibility of encoding protein is relatively low(R<1.6 or it has no open reading frame), so no protein features was predicted!		

Fig S2

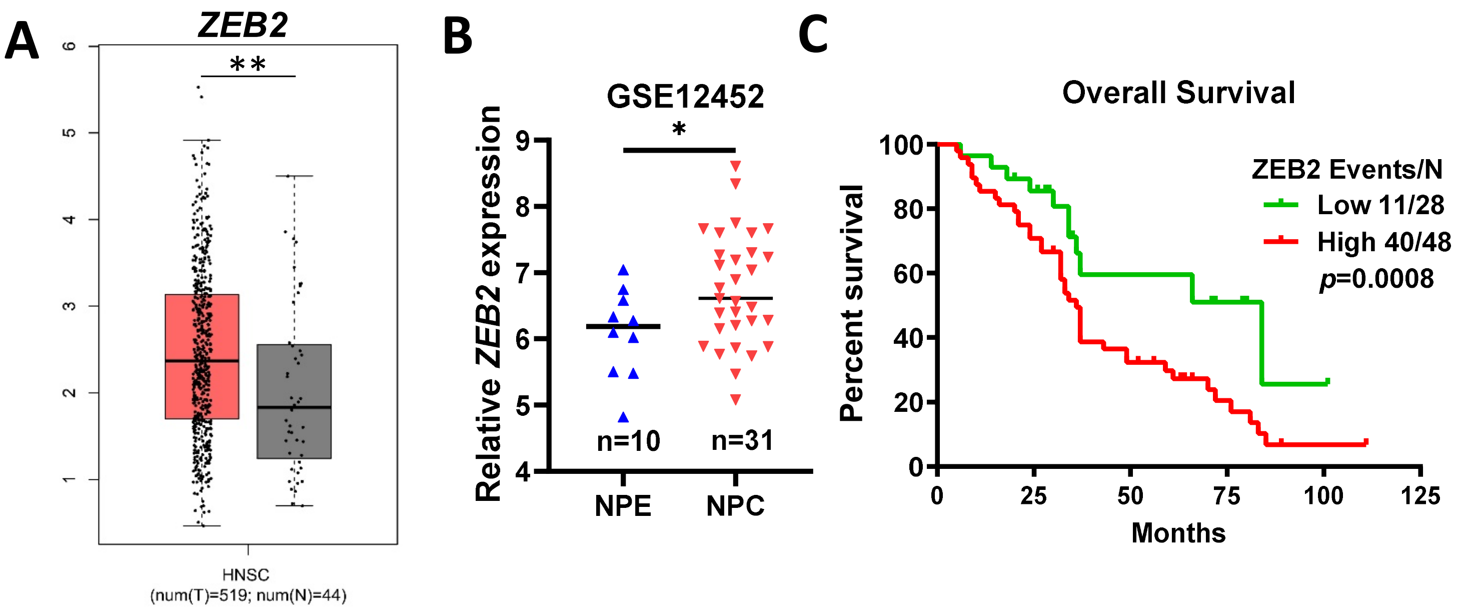

D ***ZEB2 3'UTR***

TTTCTGTACTTTTTTGTTCATTAATTTTGTCAGTATTACACCAAACTGTTTTTGCAACAAAAAAATTTTT

TTTGCATTCATTTAATTTTAGGTCAAATAACATTTTATTTATGTGGCTCATTTTTATATTTCCTAATTTTA

TTTATTTCATACTGTAGTGTACAGTATTATAGTTCTTCAATATATAGATATATTTTAGTAAAAAAGGAAC

ATGACGTTGATCATTTGGGCAAATTTTACGTAAAGAGAAGAGCATTTAATTGTGTTTTGGAACATTAATTG

TGAGATGGGATTTTTCAATTTTATTATTTTATTTTTGTTTTTTCCAATTACTGGAAATTCCAAATTTGG

Fig S3

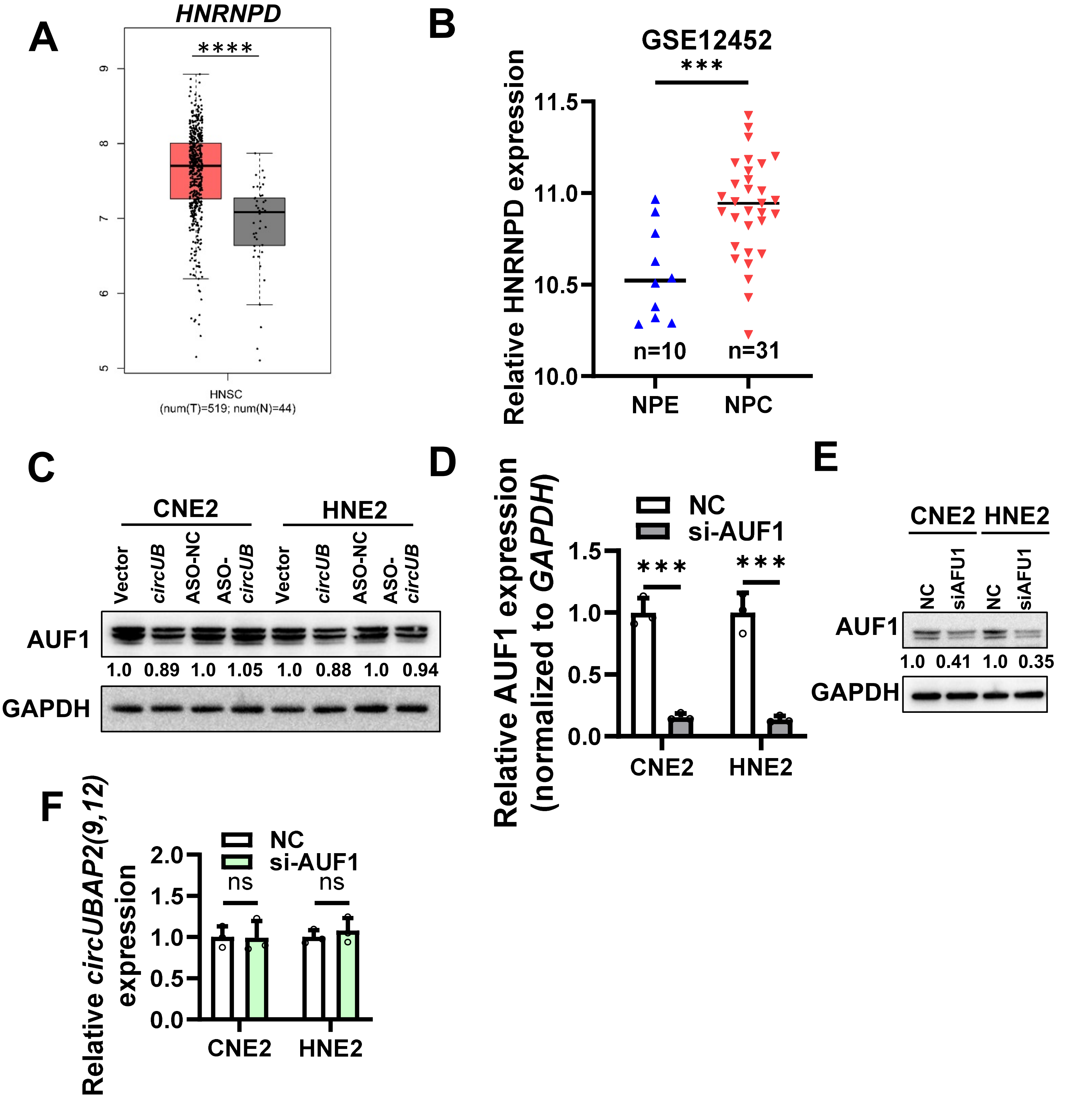

A
HNRNPD
****
HNSC
(num(T)=519; num(N)=44)
B
GSE12452
***
Relative HNRNPD expression
n=10
n=31
NPE
NPC
C
CNE2
HNE2
Vector
circUB
ASO-NC
ASO-circUB
AUF1
1.0 0.89 1.0 1.05 1.0 0.88 1.0 0.94
GAPDH
D
NC
si-AUF1
***
Relative AUF1 expression (normalized to GAPDH)
CNE2
HNE2
E
CNE2 HNE2
NC
siAFU1
AUF1
1.0 0.41 1.0 0.35
GAPDH
F
NC
si-AUF1
ns
Relative circUBAP2(9,12) expression
CNE2
HNE2

A

AUF1 *ZEB2*

#	Protein region	RNA region	Interaction Propensity	Discriminative Power	Normalized Score
1	26-77	481-578	10.93	32	3.03
3	101-152	481-578	8.33	26	2.68
4	184-235	481-578	8.01	26	2.63
6	176-227	481-578	7.38	24	2.55

AUF1 *circUBAP2(9,12)*

#	Protein region	RNA region	Interaction Propensity	Discriminative Power	Normalized Score
1	26-77	301-352	4.64	20	2.82
2	26-77	302-353	4.43	20	2.72
3	184-235	301-352	3.67	20	2.37
4	101-152	301-352	3.61	20	2.35

B

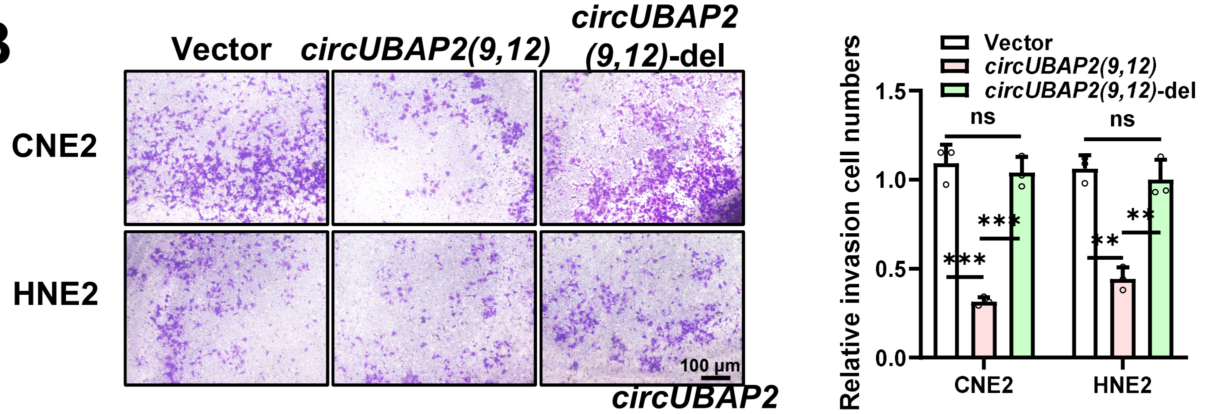

C

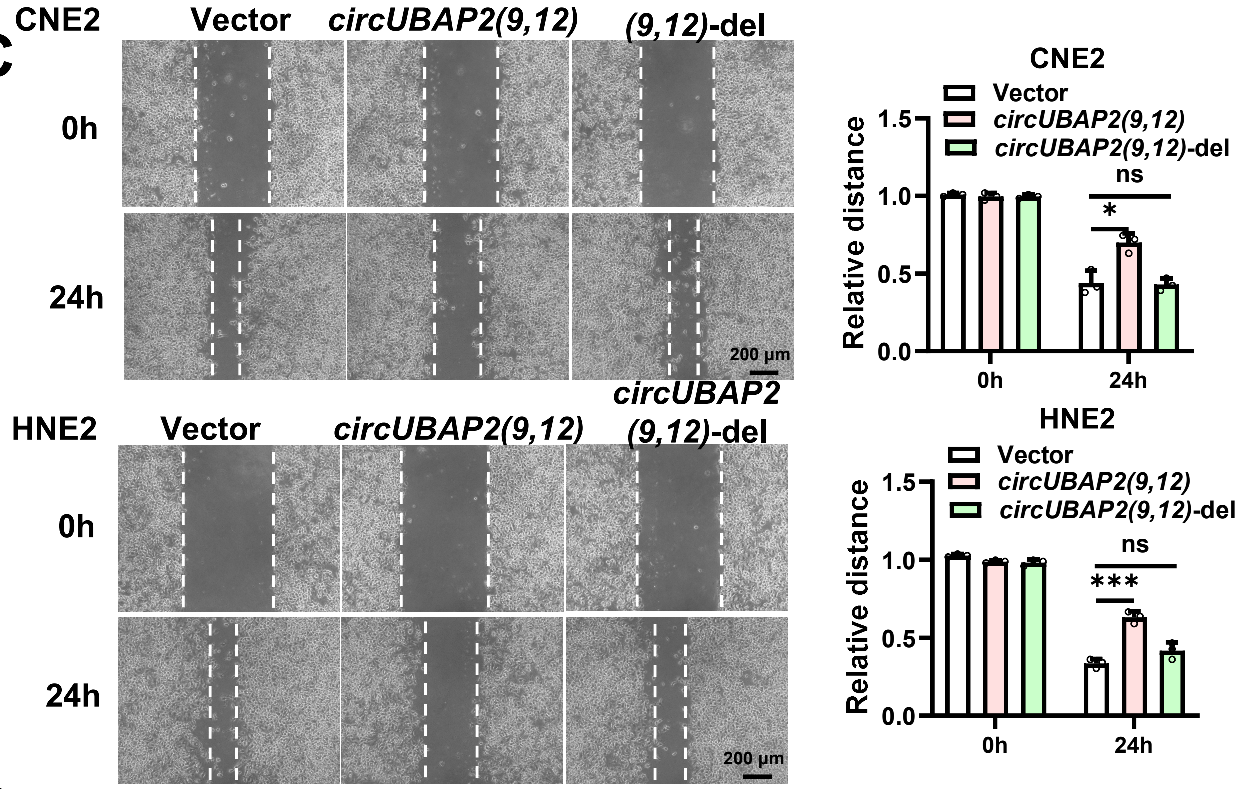

D

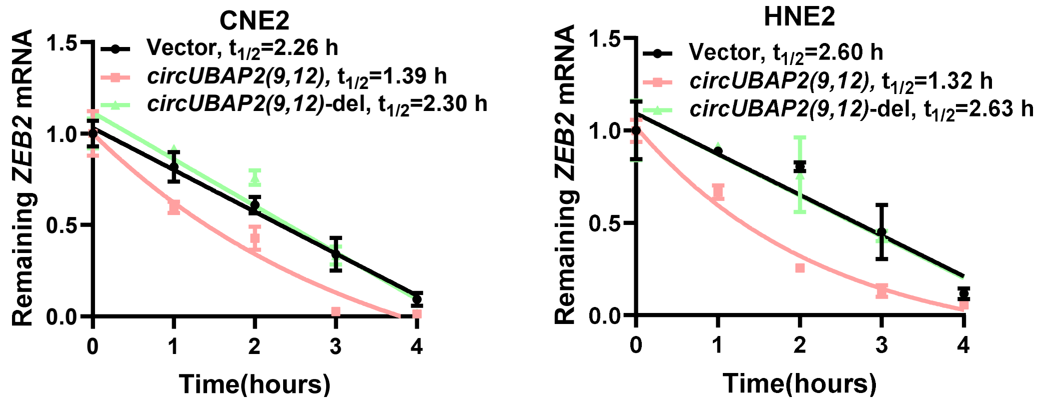

Fig S5

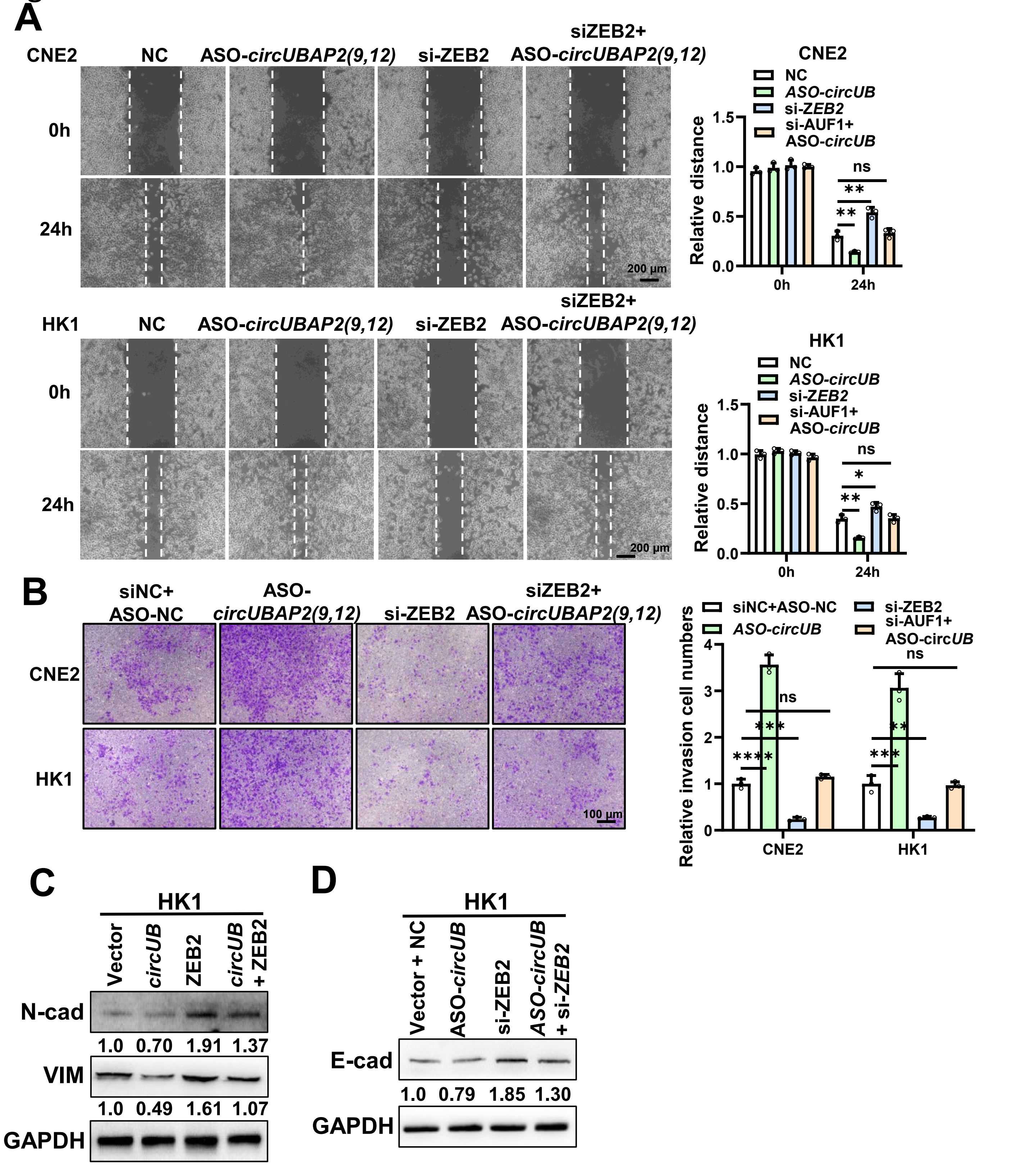

A
CNE2
NC
ASO-circUBAP2(9,12)
si-ZEB2
siZEB2+ ASO-circUBAP2(9,12)
0h
24h
200 μm
CNE2
NC
ASO-circUB
si-ZEB2
si-AUF1+ ASO-circUB
Relative distance
ns
**
0h
24h
HK1
NC
ASO-circUBAP2(9,12)
si-ZEB2
siZEB2+ ASO-circUBAP2(9,12)
0h
24h
200 μm
HK1
Relative distance
ns
*
**
B
siNC+ ASO-NC
ASO- circUBAP2(9,12)
si-ZEB2
siZEB2+ ASO-circUBAP2(9,12)
CNE2
HK1
100 μm
siNC+ASO-NC
ASO-circUB
si-ZEB2
si-AUF1+ ASO-circUB
Relative invasion cell numbers
ns
***
****
**
CNE2
HK1
C
HK1
Vector
circUB
ZEB2
circUB + ZEB2
N-cad
1.0 0.70 1.91 1.37
VIM
1.0 0.49 1.61 1.07
GAPDH
D
HK1
Vector + NC
ASO-circUB
si-ZEB2
ASO-circUB + si-ZEB2
E-cad
1.0 0.79 1.85 1.30
GAPDH
