# Supplementary material for: CircUBAP2(9,12) Inhibits Nasopharyngeal Carcinoma Invasion and Metastasis by Down-regulating ZEB2 through Competitive Binding to AUF1
Source: Research (Wash D C). 2025 Nov 4;8:0936. doi: 10.34133/research.0936 (PMC12583797; doi:10.34133/research.0936)
Supplement: Supplementary 1 — Figs. S1 to S5 Tables S1 to S7 [file research.0936.f1.zip › Supplementary Figure captions.docx]

Supplementary Figure captions


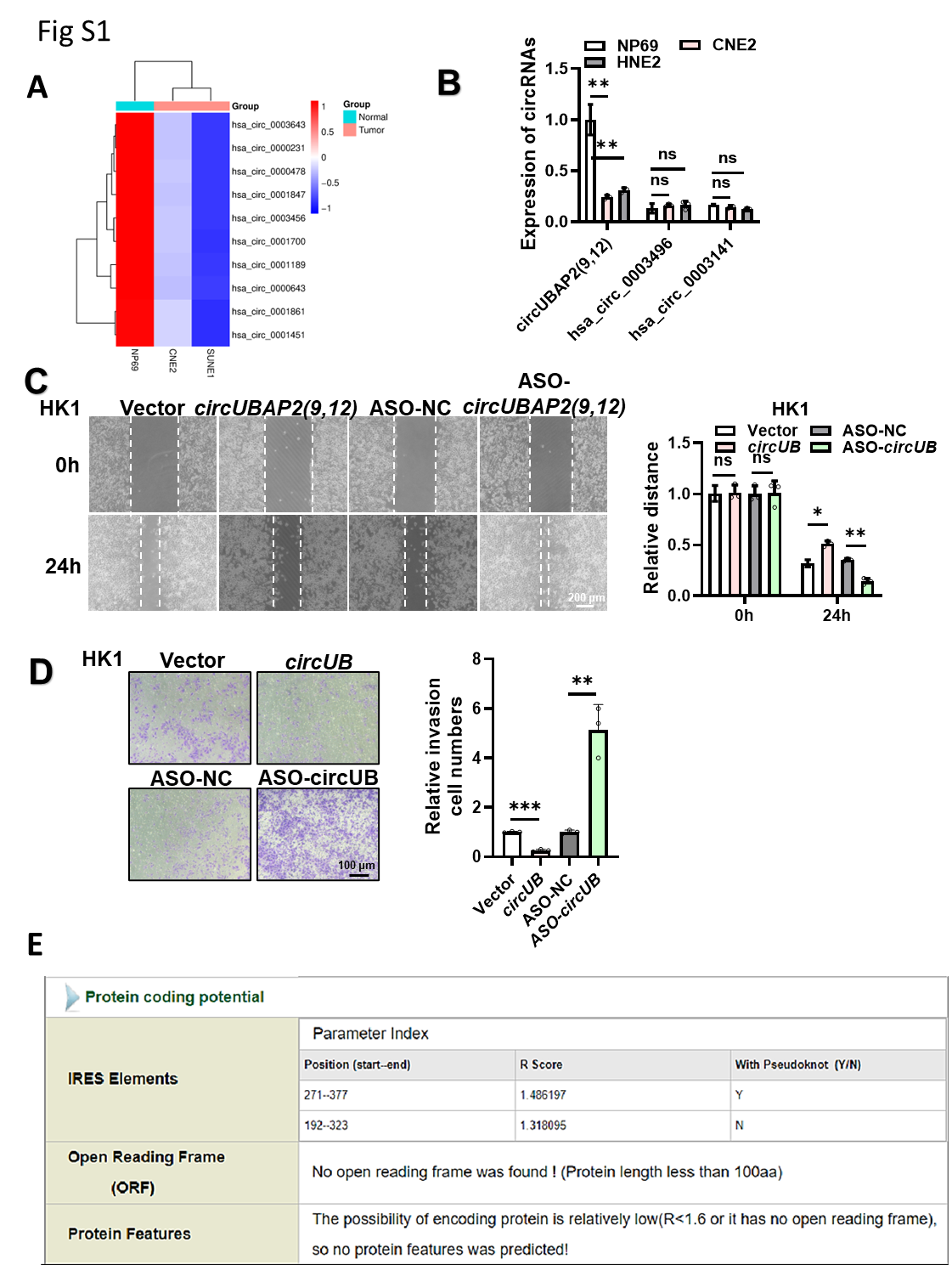


**Fig S1. *circUBAP2(9,12)* inhibits migration and invasion of NPC cells in vitro.**

(A). RNA-seq data of circRNAs from NPC cell lines CNE2 and SUNE1, as well as the normal immortalized nasopharyngeal epithelial cell line NP69 (GSE181906), were analyzed. A clustered heat map displaying the top 10 significantly downregulated circRNAs was generated using an online platform [24].

(B). The expression levels of *has_circ_0003496* (formed by circularization of exons 7-8 of *UBAP2*), *has_circ_0003141* (formed by circularization of exons 7-12 of *UBAP2*), and *circUBAP2 (9,12)* were detected by qRT-PCR in normal nasopharyngeal epithelial cell (NP69) and NPC cells (CNE2 and HNE2).

(C). The migratory capacity of HK1 cells was assessed following overexpression or knockdown of *circUBAP2(9,12)* using ImageJ by measuring residual wound area. Scale bar: 200 μm.

(D). The invasive ability of HK1 cells was evaluated after *circUBAP2(9,12)* overexpression or knockdown using transwell invasion assays. Scale bar: 100 μm.

(E). The coding potential of *circUBAP2(9,12)* was predicted using the online tool circRNAdb.

Results are shown as mean ± standard deviation from a minimum of three separate biological repetitions. ns, not significant, **p* < 0.05, ***p* < 0.01, ****p* < 0.001.


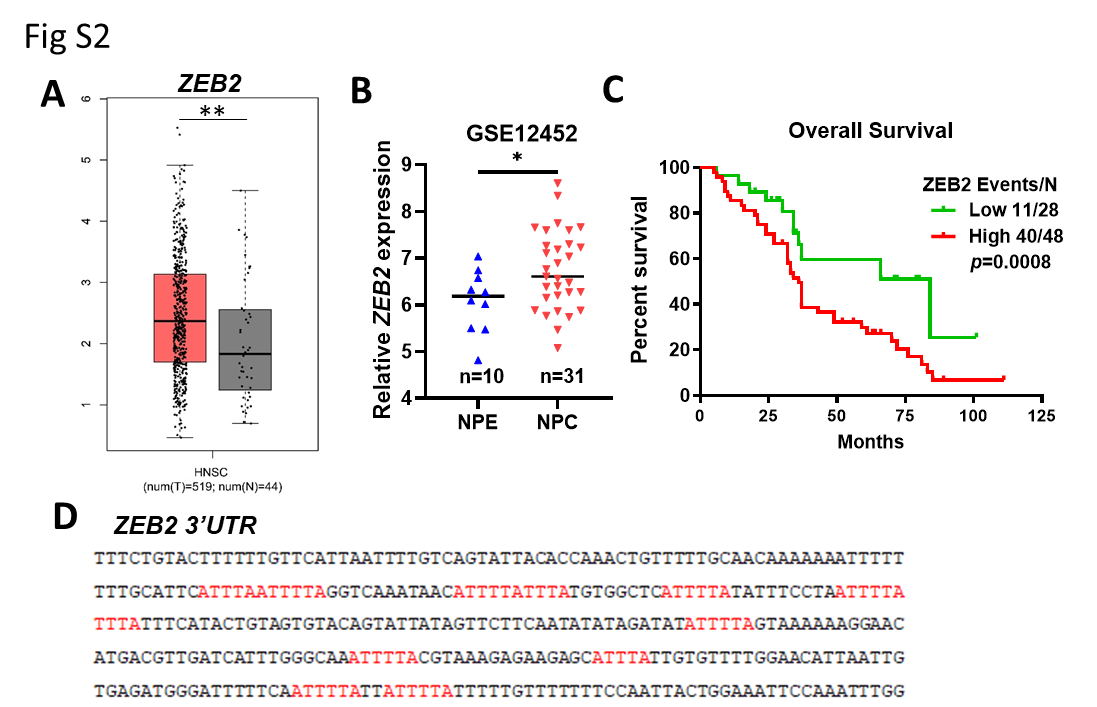


**Fig S2 ZEB2 is highly expressed in HNSC and NPC.**

(A). ZEB2 expression profiles were obtained from TCGA-HNSC cohort, comprising 519 tumor samples and 44 normal controls.

(B). Microarray analysis of GSE12452 dataset (31 NPC tissues vs. 10 normal nasopharyngeal tissues) revealed significant upregulation of ZEB2 in NPC.

(C). Kaplan–Meier survival analysis was performed for ZEB2 expression in 76 patients with NPC.

(D). *ZEB2* mRNA contains an AU-rich 3′UTR.

**p* < 0.05, ***p* < 0.01.

**
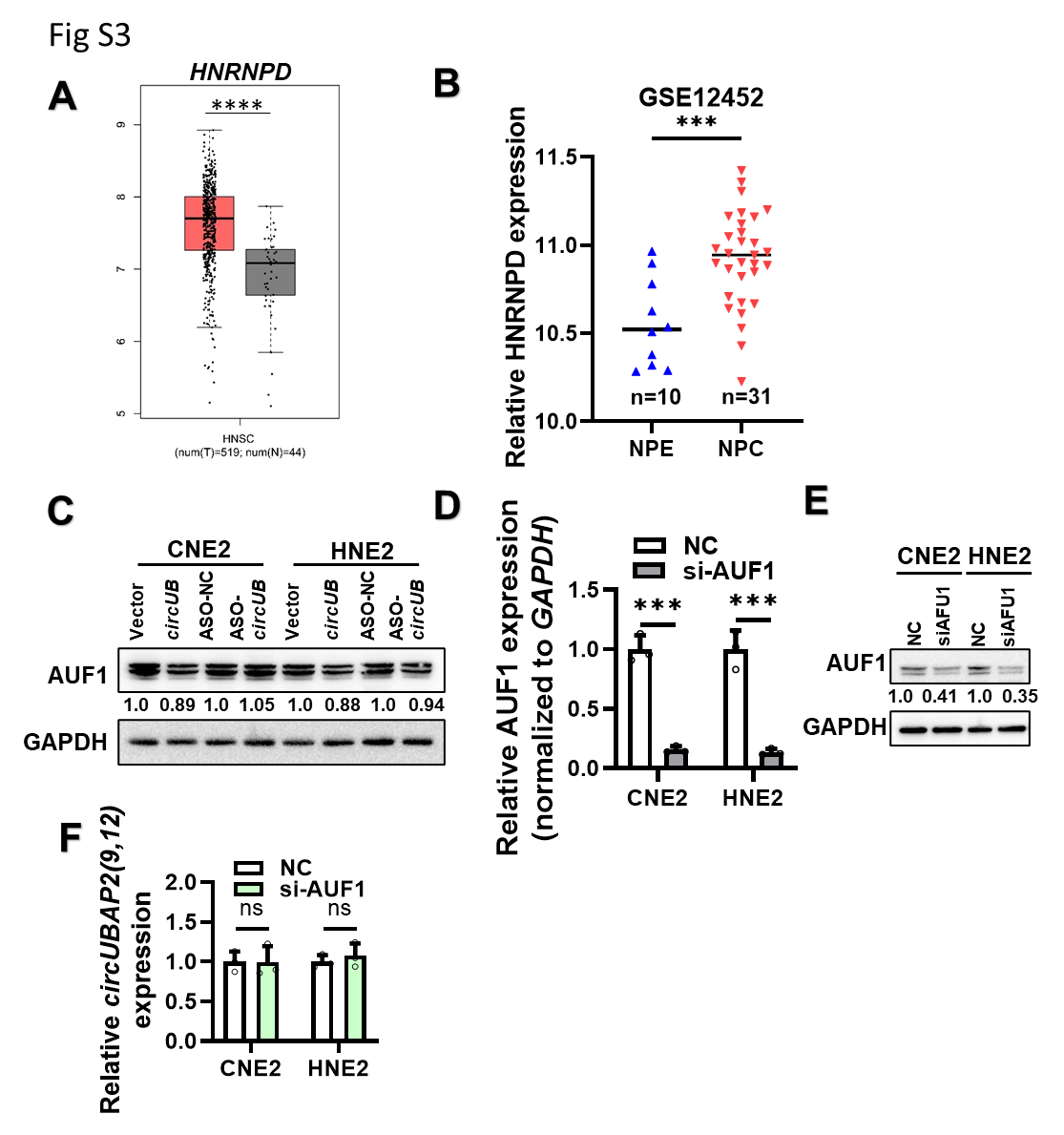
**

**Fig S3. HNRNPD is highly expressed in HNSC and NPC.**

(A). Analysis of *HNRNPD* expression in 519 HNSC tumor samples and 44 normal tissues using the TCGA database.

(B). Expression levels of *HNRNPD* in 31 NPC tissues and 10 normal nasopharyngeal tissues based on microarray data from the GSE12452 dataset.

(C). AUF1 protein levels were assessed by western blot following *circUBAP2(9,12)* overexpression or knockdown in NPC cells.

(D, E) Knockdown efficiency of AUF1 was examined by qRT-PCR (D) and western blot (E) in NPC cells.

(F). *circUBAP2(9,12)* expression was rigorously quantified by qRT-PCR after AUF1 knockdown.

Data were presented as mean ± SD from at least three independent experiments. ns, not significant, ****p* < 0.001, *****p* < 0.0001.


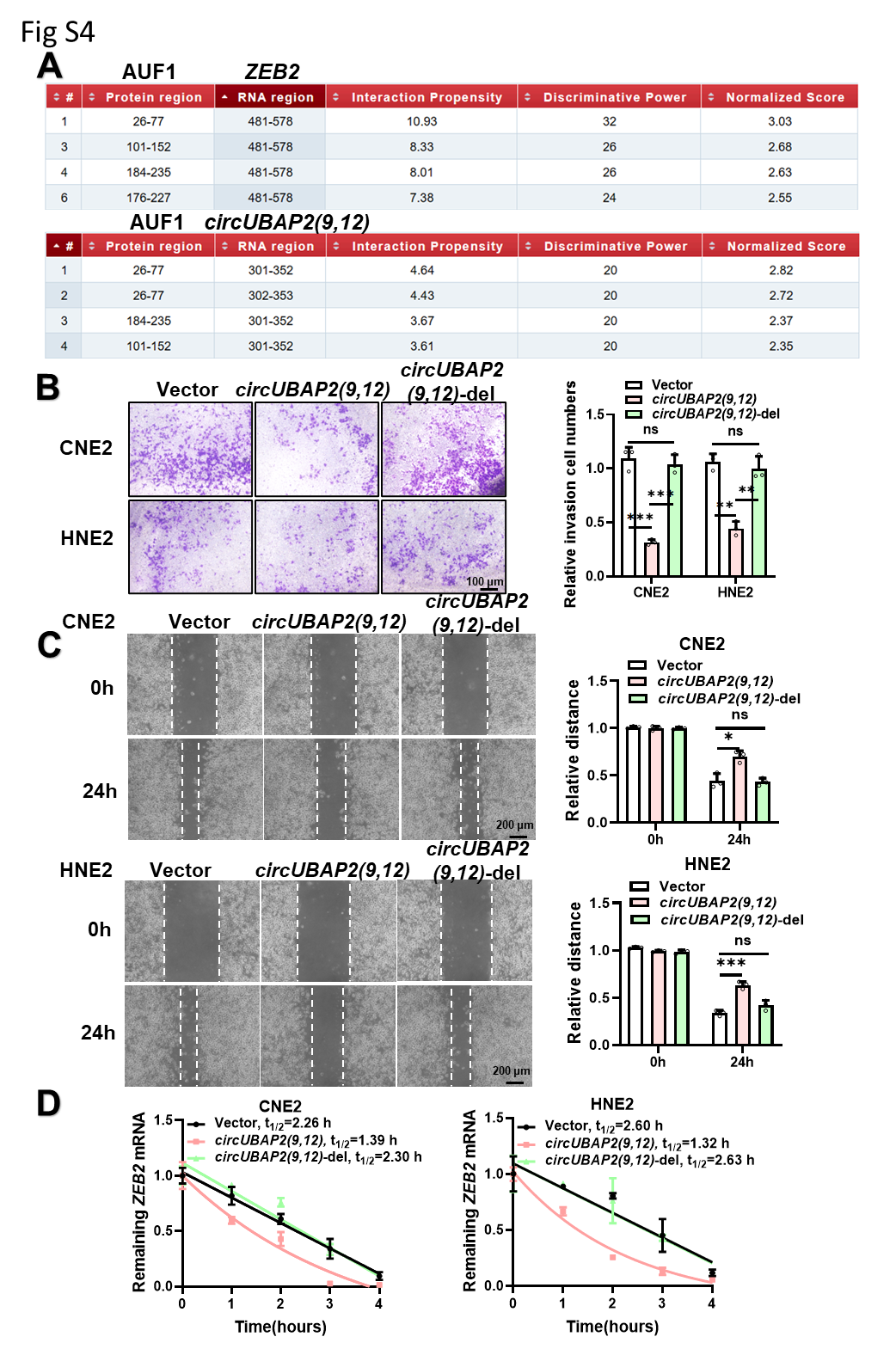


**Fig S4. *circUBAP2(9,12)*-del mutant abolished the inhibitory role of *circUBAP2(9,12)* on cell migration, invasion, and *ZEB2* mRNA stability.**

(A). RNAhybrid was used to predict the AUF1 binding regions on *circUBAP2(9,12)* and *ZEB2* mRNA.

(B). A scratch wound healing assay was performed to assess NPC cell migration after transfected with *circUBAP2(9,12)* or *circUBAP2(9,12)*-del. Scale bar: 100 μm.

(C). Transwell assay was used to quantify NPC cell invasion after transfected with *circUBAP2(9,12)* or *circUBAP2(9,12)*-del. Scale bar: 200 μm.

(D). *ZEB2* mRNA stability was assessed by actinomycin D treatment after transfection with *circUBAP2(9,12)* or *circUBAP2(9,12)*-del followed by qRT-PCR detection.

Results are shown as mean ± standard deviation from a minimum of three separate biological repetitions. ns, not significant, **p* < 0.05, ***p* < 0.01, ****p* < 0.001, *****p* < 0.0001.


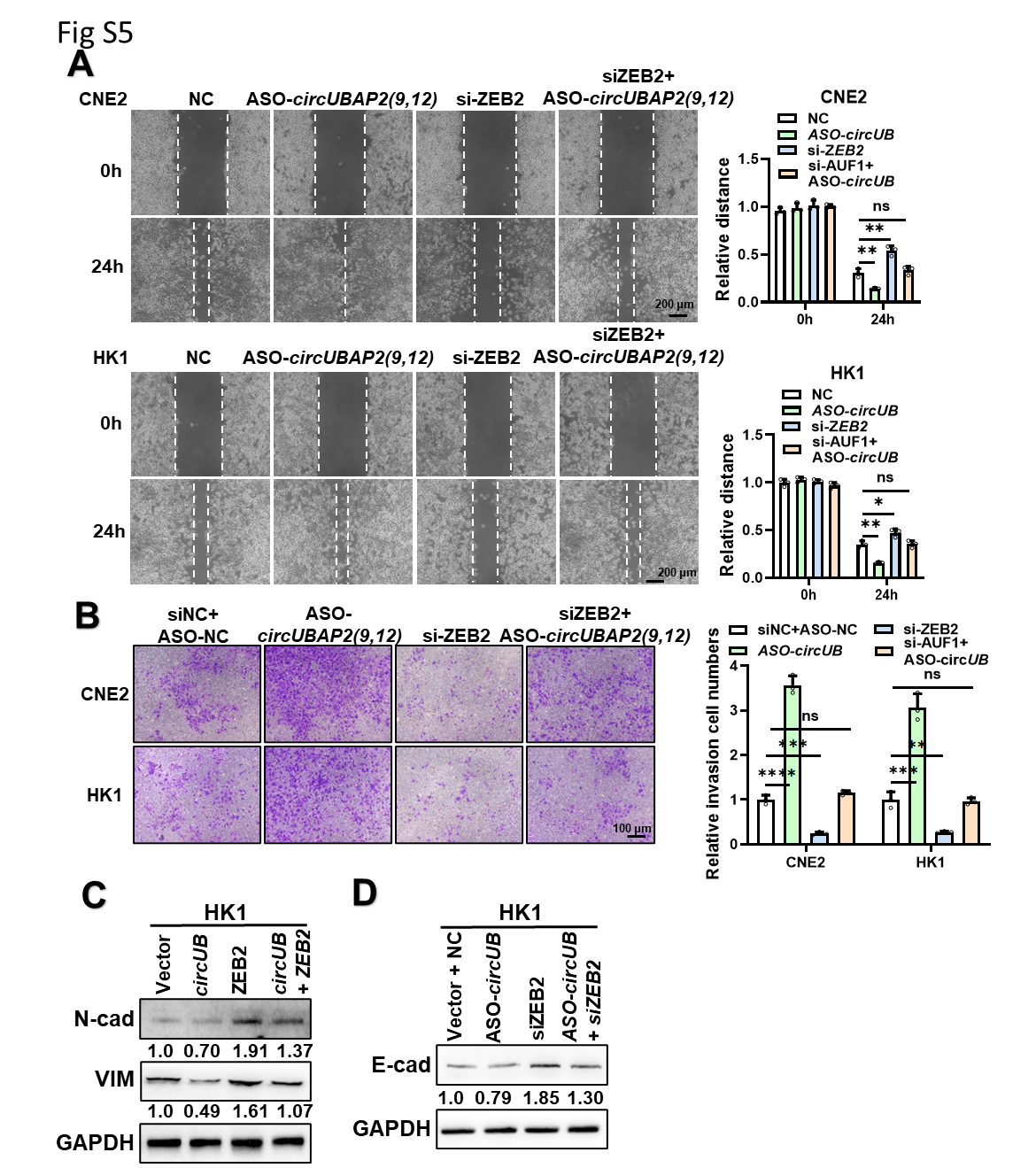


**Fig S5 *circUBAP2(9,12)* inhibits migration and invasion of NPC through ZEB2.**

(A). Wound healing assay measuring NPC cell migration after knockdown of *circUBAP2(9,12)*, ZEB2, or simultaneous knockdown of both. Scratch width was quantified using ImageJ. Scale bar = 200 μm.

(B). Transwell assay assessing NPC cell invasion following knockdown of *circUBAP2(9,12)*, ZEB2, or both. Quantification of invading cells was done using ImageJ. Scale bar = 100 μm.

(C). Western blot analysis of EMT marker after transfection with *circUBAP2(9,12)*, ZEB2, or both.

(D). Western blot analysis of E-cadherin expression after transfection with ASO-*circUBAP2(9,12)*, siZEB2, or both.

Results are shown as mean ± standard deviation from a minimum of three separate biological repetitions. ns, not significant, **p* < 0.05, ***p* < 0.01, ****p* < 0.001, *****p* < 0.0001.
