# Supplementary material for: CircUBAP2(9,12) Inhibits Nasopharyngeal Carcinoma Invasion and Metastasis by Down-regulating ZEB2 through Competitive Binding to AUF1
Source: Research (Wash D C). 2025 Nov 4;8:0936. doi: 10.34133/research.0936 (PMC12583797; doi:10.34133/research.0936)
Supplement: Supplementary 1 — Figs. S1 to S5 Tables S1 to S7 [file research.0936.f1.zip › Table S1-S6.docx]

**Table S1. ASO and siRNA sequences**

| **Target** | **Sequences (5’-3’)** |
| --- | --- |
| ASO-*circUBAP2* | CTCAGAGCCTGAACTGGCAT |
| si-AUF1 | AGCTGGGACACTACAAAGA |
| si-ZEB2 | GCACTAGTCCCTTTATGAA |

**Table S2. Clinicopathological data of 34 NPC and 8 NPE tissues** **used for qRT-PCR**

| Patient No. | Gender (M=Male F=Female) | WHO histological diagnosis | T | N | M | Clinical Stages |
| --- | --- | --- | --- | --- | --- | --- |
| N1 | M | non-tumor nasopharyngeal epithelial | NA | NA | NA | NA |
| N2 | M | non-tumor nasopharyngeal epithelial | NA | NA | NA | NA |
| N3 | M | non-tumor nasopharyngeal epithelial | NA | NA | NA | NA |
| N4 | F | non-tumor nasopharyngeal epithelial | NA | NA | NA | NA |
| N5 | M | non-tumor nasopharyngeal epithelial | NA | NA | NA | NA |
| N6 | F | non-tumor nasopharyngeal epithelial | NA | NA | NA | NA |
| N7 | F | non-tumor nasopharyngeal epithelial | NA | NA | NA | NA |
| N8 | M | non-tumor nasopharyngeal epithelial | NA | NA | NA | NA |
| T1 | M | nasopharyngeal squamous cell carcinoma | 2 | 1 | 0 | Ib |
| T2 | F | nasopharyngeal squamous cell carcinoma | 1 | 1 | 0 | Ib |
| T3 | M | nasopharyngeal squamous cell carcinoma | 3 | 2 | 0 | II |
| T4 | M | nasopharyngeal squamous cell carcinoma | 2 | 2 | 0 | II |
| T5 | M | nasopharyngeal squamous cell carcinoma | 3 | 2 | 0 | II |
| T6 | F | nasopharyngeal squamous cell carcinoma | 3 | 2 | 0 | II |
| T7 | F | nasopharyngeal squamous cell carcinoma | 3 | 2 | 0 | II |
| T8 | F | nasopharyngeal squamous cell carcinoma | 3 | 2 | 0 | II |
| T9 | M | nasopharyngeal squamous cell carcinoma | 4 | 2 | 0 | III |
| T10 | M | nasopharyngeal squamous cell carcinoma | 2 | 3 | 0 | III |
| T11 | F | nasopharyngeal squamous cell carcinoma | 4 | 3 | 0 | III |
| T12 | M | nasopharyngeal squamous cell carcinoma | 2 | 3 | 0 | III |
| T13 | F | nasopharyngeal squamous cell carcinoma | 4 | 3 | 0 | III |
| T14 | M | nasopharyngeal squamous cell carcinoma | 4 | 3 | 0 | III |
| T15 | M | nasopharyngeal squamous cell carcinoma | 4 | 3 | 1a | IVa |
| T16 | F | nasopharyngeal squamous cell carcinoma | 3 | 3 | 0 | III |
| T17 | M | nasopharyngeal squamous cell carcinoma | 2 | 3 | 0 | III |
| T18 | F | nasopharyngeal squamous cell carcinoma | 3 | 3 | 0 | III |
| T19 | F | nasopharyngeal squamous cell carcinoma | 2 | 3 | 0 | III |
| T20 | F | nasopharyngeal squamous cell carcinoma | 3 | 3 | 0 | III |
| T21 | M | nasopharyngeal squamous cell carcinoma | 3 | 3 | 0 | III |
| T22 | F | nasopharyngeal squamous cell carcinoma | 2 | 3 | 0 | III |
| T23 | F | nasopharyngeal squamous cell carcinoma | 2 | 3 | 0 | III |
| T24 | M | nasopharyngeal squamous cell carcinoma | 4 | 1 | 0 | III |
| T25 | M | nasopharyngeal squamous cell carcinoma | 4 | 1 | 0 | III |
| T26 | M | nasopharyngeal squamous cell carcinoma | 3 | 3 | 0 | III |
| T27 | M | nasopharyngeal squamous cell carcinoma | 3 | 3 | 0 | III |
| T28 | M | nasopharyngeal squamous cell carcinoma | 2 | 3 | 0 | III |
| T29 | M | nasopharyngeal squamous cell carcinoma | 2 | 3 | 0 | III |
| T30 | M | nasopharyngeal squamous cell carcinoma | 3 | 3 | 0 | III |
| T31 | F | nasopharyngeal squamous cell carcinoma | 3 | 3 | 1a | IVa |
| T32 | M | nasopharyngeal squamous cell carcinoma | 3 | 3 | 1a | IVa |
| T33 | M | nasopharyngeal squamous cell carcinoma | 3 | 2 | 1b | IVb |
| T34 | M | nasopharyngeal squamous cell carcinoma | 1 | 3 | 1b | IVb |
| NPC = nasopharyngeal carcinoma; NPE = nontumor nasopharyngeal epithelium | | | | | | |
| Clinical stages are scored according to Ninth Version of the AJCC and UICC Nasopharyngeal Cancer TNM Staging Classification | | | | | | |

**Table S3. Clinicopathological data of 76 paraffin-embedded NPC tissues used for ISH**

| **Patient No.** | **Gender (M=Male F=Female)** | **Age at Diagnosis** | **T stage** | **N stage** | **M stage** | **Clinical stages** | **OS time (Months)** | **Over-all survival (0=alive, 1=death)** |
| --- | --- | --- | --- | --- | --- | --- | --- | --- |
| T1 | M | 55 | 2 | 2 | 0 | II | 14 | 1 |
| T2 | F | 44 | 3 | 1 | 0 | II | 6 | 1 |
| T3 | M | 52 | 4 | 1 | 0 | III | 101 | 1 |
| T4 | M | 50 | 4 | 3 | 0 | III | 66 | 1 |
| T5 | M | 50 | 1 | 0 | 0 | Ia | 84 | 1 |
| T6 | M | 55 | 2 | 3 | 0 | III | 9 | 1 |
| T7 | M | 36 | 2 | 2 | 0 | II | 32 | 1 |
| T8 | M | 56 | 3 | 3 | 0 | III | 76 | 1 |
| T9 | M | 61 | 2 | 0 | 0 | Ia | 111 | 1 |
| T10 | M | 56 | 3 | 3 | 0 | III | 70 | 1 |
| T11 | M | 76 | 4 | 0 | 0 | III | 21 | 1 |
| T12 | M | 50 | 3 | 2 | 0 | II | 59 | 1 |
| T13 | M | 46 | 3 | 3 | 0 | III | 61 | 1 |
| T14 | M | 1 | 3 | 3 | 0 | III | 85 | 1 |
| T15 | M | 38 | 4 | 2 | 1a | IVa | 20 | 1 |
| T16 | F | 43 | 1 | 3 | 1a | IVa | 32 | 1 |
| T17 | M | 37 | 2 | 3 | 0 | III | 89 | 0 |
| T18 | M | 54 | 2 | 1 | 1a | IVa | 83 | 1 |
| T19 | M | 31 | 2 | 2 | 0 | II | 49 | 1 |
| T20 | F | 52 | 1 | 0 | 0 | Ia | 49 | 1 |
| T21 | M | 35 | 2 | 2 | 0 | II | 64 | 1 |
| T22 | F | 55 | 3 | 2 | 1b | Ivb | 10 | 1 |
| T23 | M | 51 | 2 | 2 | 0 | II | 8 | 1 |
| T24 | M | 44 | 1 | 1 | 0 | Ib | 15 | 1 |
| T25 | M | 38 | 3 | 0 | 0 | II | 5 | 1 |
| T26 | M | 39 | 2 | 2 | 0 | II | 32 | 1 |
| T27 | M | 38 | 3 | 2 | 0 | II | 24 | 1 |
| T28 | F | 51 | 2 | 2 | 0 | II | 16 | 1 |
| T29 | M | 49 | 4 | 2 | 0 | III | 11 | 1 |
| T30 | M | 44 | 2 | 2 | 0 | II | 27 | 1 |
| T31 | F | 60 | 4 | 2 | 0 | III | 37 | 0 |
| T32 | F | 39 | 4 | 2 | 0 | III | 9 | 1 |
| T33 | F | 40 | 2 | 2 | 0 | II | 28 | 0 |
| T34 | M | 39 | 2 | 0 | 0 | Ia | 37 | 0 |
| T35 | M | 50 | 4 | 2 | 0 | III | 36 | 0 |
| T36 | M | 45 | 3 | 0 | 0 | II | 36 | 0 |
| T37 | M | 41 | 4 | 2 | 0 | III | 33 | 0 |
| T38 | F | 46 | 4 | 1 | 0 | III | 33 | 0 |
| T39 | F | 45 | 3 | 0 | 0 | II | 32 | 0 |
| T40 | M | 45 | 3 | 2 | 0 | II | 6 | 1 |
| T41 | F | 62 | 2 | 2 | 0 | II | 30 | 1 |
| T42 | F | 36 | 2 | 2 | 0 | II | 34 | 1 |
| T43 | M | 49 | 2 | 3 | 0 | III | 34 | 1 |
| T44 | M | 43 | 2 | 2 | 0 | II | 36 | 0 |
| T45 | M | 57 | 3 | 2 | 0 | II | 24 | 1 |
| T46 | M | 57 | 3 | 2 | 0 | II | 24 | 1 |
| T47 | M | 44 | 2 | 2 | 0 | II | 27 | 1 |
| T48 | M | 39 | 4 | 1 | 0 | III | 37 | 0 |
| T49 | M | 39 | 4 | 1 | 0 | III | 37 | 1 |
| T50 | F | 47 | 1 | 3 | 0 | III | 36 | 1 |
| T51 | M | 60 | 1 | 1 | 0 | Ib | 21 | 1 |
| T52 | M | 66 | 2 | 2 | 0 | II | 80 | 1 |
| T53 | F | 41 | 2 | 1 | 0 | Ib | 81 | 0 |
| T54 | M | 58 | 3 | 0 | 0 | II | 71 | 1 |
| T55 | M | 52 | 1 | 2 | 0 | II | 79 | 1 |
| T56 | M | 64 | 2 | 0 | 0 | Ia | 72 | 1 |
| T57 | M | 64 | 2 | 0 | 0 | Ia | 72 | 0 |
| T58 | F | 38 | 2 | 1 | 0 | Ib | 43 | 0 |
| T59 | M | 23 | 3 | 2 | 0 | II | 37 | 0 |
| T60 | M | 68 | 2 | 1 | 0 | Ib | 52 | 0 |
| T61 | M | 70 | 2 | 2 | 0 | II | 66 | 0 |
| T62 | M | 42 | 2 | 3 | 0 | III | 63 | 0 |
| T63 | M | 36 | 2 | 2 | 0 | II | 20 | 1 |
| T64 | M | 62 | 1 | 0 | 0 | Ia | 18 | 1 |
| T65 | M | 22 | 3 | 1 | 0 | II | 35 | 1 |
| T66 | M | 63 | 3 | 2 | 1b | IVb | 34 | 1 |
| T67 | M | 76 | 4 | 0 | 0 | III | 24 | 1 |
| T68 | M | 61 | 2 | 2 | 0 | II | 37 | 1 |
| T69 | M | 66 | 4 | 0 | 0 | III | 26 | 1 |
| T70 | F | 46 | 4 | 1 | 0 | III | 28 | 1 |
| T71 | F | 59 | 3 | 2 | 0 | II | 29 | 1 |
| T72 | M | 61 | 2 | 2 | 0 | II | 37 | 0 |
| T73 | M | 47 | 2 | 3 | 1a | IVa | 56 | 0 |
| T74 | M | 43 | 1 | 3 | 1a | IVa | 36 | 1 |
| T75 | M | 61 | 2 | 2 | 0 | II | 37 | 1 |
| T76 | M | 63 | 3 | 2 | 1a | IVa | 30 | 0 |

Clinical stages are scored according to Ninth Version of the AJCC and UICC Nasopharyngeal Cancer TNM Staging Classification.

**Table S4.** **Probe sequences for RNA pulldown, FISH and ISH**

| **Probe name** | **Sequences (5’-3’)** |
| --- | --- |
| *CircUBAP2*-biotin | biotin-TATTTGATGCCAGTTCAGGCTCTGAGGAGA-biotin |
| *CircUBAP2*-unbiotin  *CircUBAP2*-digoxin | TATTTGATGCCAGTTCAGGCTCTGAGGAGA  digoxin-TATTTGATGCCAGTTCAGGCTCTGAGGAGA-digoxin |

**Table S5. Primer sequences for qRT-PCR**

| **Target** | **Sequences (5’-3’)** |
| --- | --- |
| *CircUBAP2*-F | TGAAACTTCCCAACAGCAGG |
| *CircUBAP2*-R | TGTTGTCCACTCTTCCACAGA |
| *AUF1*-F | CTAGACAGACAGCAACGGGA |
| *AUF1*-R | GCGTAATGGCATAGGTGGTG |
| *UBAP2*-F | CCCCGCGAGTGCATCCAGCA |
| *UBAP2*-R | CGGCTGGCAAGCGCTGTGGG |
| *GAPDH*-F | CAAGGTCATCCATGACAACTTTG |
| *GAPDH*-R | GTCCACCACCCTGTTGCTGTAG |
| *18S*-F | TCTTAGCTGAGTGTCCCGCG |
| *18S*-R | ATCATGGCCTCAGTTCCGAA |
| *β-actin*-F | TCACCAACTGGGACGACATG |
| *β-actin*-R | GTCACCGGAGTCCATCACGAT |
| *U6*-F | CTCGCTTCGGCAGCACA |
| *U6*-R | AACGCTTCACGAATTTGCGT |
| *ZEB2*-F | CAAGAGGCGCAAACAAGCC |
| *ZEB2*-R | GGTTGGCAATACCGTCATCC |
| *ZEB1*-F | GATGATGAATGCGAGTCAGATGC |
| *ZEB1*-R | ACAGCAGTGTCTTGTTGTTGT |
| *SNAIL2*-F | CGAACTGGACACACATACAGTG |
| *SNAIL2*-R | CTGAGGATCTCTGGTTGTGGT |
| *SNAIL1*-F | TCGGAAGCCTAACTACAGCGA |
| *SNAIL1*-R | AGATGAGCATTGGCAGCGAG |
| *TWIST1*-F | GTCCGCAGTCTTACGAGGAG |
| *TWIST1*-R | GCTTGAGGGTCTGAATCTTGCT |
| *VIM*-F | CGCCAACTACATCGACAAGG |
| *VIM*-R | GGCTTTGTCGTTGGTTAGCT |
| *CDH1*-F | ATGAAGAAGGAGGCGGAGAAGAGG |
| *CDH1*-R | TGCAACGTCGTTACGAGTCACTTC |
| *CDH2*-F | AGCACAGTGGCCACCTACAAAG |
| *CDH2*-R | CAGCTCCTGGCCCAGTTACA |

**Table S6. List of antibodies used**

| **Antibody** | **Catalog** | **Company** |
| --- | --- | --- |
| AUF1 Rabbit antibody | 19887- 1-AP | Proteintech |
| Anti-rabbit IgG, HRP-linked Antibody | 7074P2 | Cell Signaling Technology |
| Anti-mouse IgG, HRP-linked Antibody | 7076P2 | Cell Signaling Technology |
| Anti-β-Tubulin Mouse Antibody | A01030 | Abbkine |
| Vimentin Recombinant Rabbit Monoclonal Antibody | AWA10146 | Abiowell |
| E-Cadherin Mouse Monoclonal Antibody  N-Cadherin (D4R1H) XP® Rabbit mAb | AWA01746  #13116 | Abiowell  Cell Signaling Technology |
| SIP1(ZEB2) Polyclonal Antibody | YT4300 | ImmunoWay Biotechnology |
